# Machine learning applications for therapeutic tasks with genomics data

**DOI:** 10.1016/j.patter.2021.100328

**Published:** 2021-08-09

**Authors:** Kexin Huang, Cao Xiao, Lucas M. Glass, Cathy W. Critchlow, Greg Gibson, Jimeng Sun

**Affiliations:** 1 Department of Computer Science, Stanford University, Stanford, CA 94305, USA; 2 Amplitude, San Francisco, CA 94105, USA; 3 Analytics Center of Excellence, IQVIA, Cambridge, MA 02139, USA; 4 Center for Observational Research, Amgen, Thousand Oaks, CA 91320, USA; 5 Center for Integrative Genomics, Georgia Institute of Technology, Atlanta, GA 30332, USA; 6 Computer Science Department and Carle's Illinois College of Medicine, University of Illinois at Urbana-Champaign, Urbana, IL 61820, USA

**Keywords:** machine learning, therapeutics discovery and development, genomics

## Abstract

Thanks to the increasing availability of genomics and other biomedical data, many machine learning algorithms have been proposed for a wide range of therapeutic discovery and development tasks. In this survey, we review the literature on machine learning applications for genomics through the lens of therapeutic development. We investigate the interplay among genomics, compounds, proteins, electronic health records, cellular images, and clinical texts. We identify 22 machine learning in genomics applications that span the whole therapeutics pipeline, from discovering novel targets, personalizing medicine, developing gene-editing tools, all the way to facilitating clinical trials and post-market studies. We also pinpoint seven key challenges in this field with potentials for expansion and impact. This survey examines recent research at the intersection of machine learning, genomics, and therapeutic development.

## The bigger picture

The genome contains instructions for building the function and structure of organisms. Recent high-throughput techniques have made it possible to generate massive amounts of genomics data. However, there are numerous roadblocks on the way to turning genomic data into tangible therapeutics. We observe that genomics data alone are insufficient for therapeutic development. We need to investigate how genomics data interact with other types of data such as compounds, proteins, electronic health records, images, and texts. Machine learning techniques can be used to identify patterns and extract insights from these complex data. In this review, we survey a wide range of genomics applications of machine learning that can enable faster and more efficacious therapeutic development. Challenges remain, including technical problems such as learning under different contexts given low-resource constraints, and practical issues such as mistrust of models, privacy, and fairness.

## Teaser

Recent high-throughput techniques have made it possible to generate massive amounts of genomics data. However, there are numerous roadblocks on the way to turning genomic data into tangible therapeutics. We need to investigate how genomics data interact with other types of data such as compounds, proteins, electronic health records, images, and texts. In this review, we survey a wide range of genomics applications of machine learning that can enable faster and more efficacious therapeutic development.

## Introduction

Genomics studies the function, structure, evolution, mapping, and editing of genomes.^1^ It allows us to understand biological phenomena, such as the roles that the genome plays in diseases. A deep understanding of genomics has led to a vast array of successful therapeutics to cure a wide range of diseases, both complex and rare.^2^^,^^3^ It also allows us to prescribe more precise treatments^4^ or seek more effective therapeutics strategies such as genome editing.^5^

Recent advances in high-throughput technologies have led to an outpouring of large-scale genomics data.^6^^,^^7^ However, the bottlenecks along the path of transforming genomics data into tangible therapeutics are innumerable. For instance, diseases are driven by multifaceted mechanisms, so to pinpoint the right disease target requires knowledge about the entire suite of biological processes, including gene regulation by non-coding regions,^8^ DNA methylation status,^9^ and RNA splicing;^10^ personalized treatment requires accurate characterization of disease subtypes, and the compound's sensitivity to various genomics profiles;^4^ gene-editing tools require an understanding of the interplay between guide RNA and the whole-genome to avoid off-target effects;^11^ monitoring therapeutics efficacy and safety after approval requires the mining of gene-drug-disease relations in the electronic health record (EHR) and literature.^12^ We argue that genomics data alone are insufficient to ensure clinical implementation, but it requires the integration of a diverse set of data types, from compounds, proteins, cellular image, and EHRs to scientific literature. This heterogeneity and scale of data enable the application of sophisticated computational methods such as machine learning (ML).

Over the years, ML has profoundly impacted many application domains, such as computer vision,^13^ natural language processing,^14^ and complex systems.^15^ ML has changed computational modeling from expert-curated features to automated feature construction. It can learn useful and novel patterns from data, often not found by experts, to improve prediction performance on various tasks. This ability is much needed in genomics and therapeutics, as our understanding of human biology is vastly incomplete. Uncovering these patterns can also lead to the discovery of novel biological insights. Also, therapeutic discovery often consists of large-scale resource-intensive experiments, which limit the scope of experiments, and many potent candidates are therefore missed. Using accurate prediction by ML can drastically scale up and facilitate the experiments, catching or generating novel therapeutics candidates.

Interests in ML for genomics through the lens of therapeutic development have also grown for two reasons. First, for pharmaceutical and biomedical researchers, ML models have undergone proof-of-concept stages in yielding astounding performance often for previously infeasible tasks.^16^^,^^17^ Second, for ML scientists, large/complex data and difficult/impactful problems present exciting opportunities for innovation.

This survey summarizes recent ML applications related to genomics in therapeutic development and describes associated challenges and opportunities. We broadly define the term genomics as functional aspects of genes, including what genes are present, how genes are expressed given different contexts, what the relations are among genes, and so forth. Several reviews of ML for genomics have been published.^18^, ^19^, ^20^ Most of these previous works focused on studying genomics for biological applications, whereas we study them in the context of bringing genomics discovery to therapeutic implementations. We identify 22 “ML for therapeutics” tasks with genomics data, ranging across the entire therapeutic pipeline, which was not covered in previous surveys. Moreover, most of the previous reviews focused on DNA sequences, while we go beyond DNA sequences and study a wide range of interactions among DNA sequences, compounds, proteins, multi-omics, and EHR data.

In this survey, we organize ML applications into four therapeutic pipelines: (1) target discovery: basic biomedical research to discover novel disease targets to enable therapeutics; (2) therapeutic discovery: large-scale screening designed to identify potent and safe therapeutics; (3) clinical study: evaluating the efficacy and safety of the therapeutics *in vitro*, *in vivo*, and through clinical trials; and (4) post-market study: monitoring the safety and efficacy of marketed therapeutics and identifying novel indications. We also formulate these tasks and data modalities in ML languages, which can help ML researchers with limited domain background to understand those tasks. In summary, this survey presents a unique perspective on the intersection of ML, genomics, and therapeutic development.

**Figure 1:**
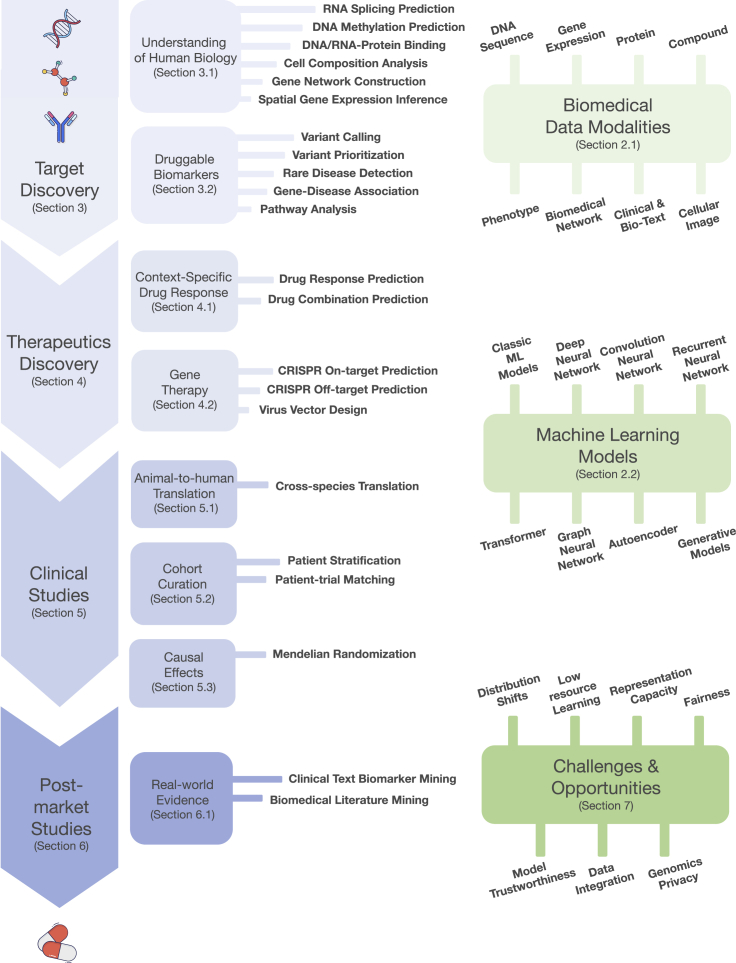
Organization and coverage of this survey Our survey covers a wide range of important ML applications in genomics across the therapeutics pipelines. In addition, we provide a primer on biomedical data modalities and machine learning models. Finally, we identify seven challenges filled with opportunities.

The survey is organized as follows (Figure 1). In the next section, we provide a brief primer on genomics-related data. We also review popular ML models for each data type. In the subsequent three sections, we discuss ML applications in genomics across the therapeutics development pipeline. Each section describes a phase in the therapeutics pipeline and contains several ML applications and ML models and formulations. In the penultimate section, we identify seven open challenges that present numerous opportunities for ML model development and also novel applications. We provide a GitHub repository (https://github.com/kexinhuang12345/ml-genomics-resources) that curates a list of resources discussed in this survey.

## A primer on genomics data and machine learning models

With advances in high-throughput technologies and data management systems, we now have vast and heterogeneous datasets in the field of biomedicine. This section introduces the basic genomics-related data types and their ML representation and provides a primer on popular ML methods applied to these data. First, we discuss the data representatsion in genomics-related tasks. In Table 1, we provide a set of pointers to high-quality datasets that cover the discussed data representations.

**Table 1:** High-quality machine learning datasets references and pointers for genomics therapeutics tasks

Pipeline (related section)	Task	Reference	Data link
Target discovery (“machine learning for genomics in target discovery”)	DNA/RNA-protein binding	Zeng et al.^57^	http://cnn.csail.mit.edu/
	methylation state	Levy et al.^195^	https://github.com/Christensen-Lab-Dartmouth/PyMethylProcess
	RNA splicing	Harrow et al.^196^	https://www.gencodegenes.org/
	spatial gene expression	Weinstein et al.^197^	https://portal.gdc.cancer.gov/
	cell-composition analysis	Avila Cobos et al.^198^	https://go.nature.com/3mxCZEv
	gene network construction	Shrivastava et al.^86^	https://github.com/Harshs27/GRNUlar
	variant calling	Chen et al.^199^	https://www.ncbi.nlm.nih.gov/bioproject/PRJNA511646/
	variant prioritization	Landrum et al.^200^	https://www.ncbi.nlm.nih.gov/clinvar/
	gene-disease association	Piñero et al.^201^	https://www.disgenet.org/
	pathway analysis	Fabregat et al.^202^	https://reactome.org/
Therapeutics discovery (“machine learning for genomics in therapeutics discovery”	drug response	Yang et al.^203^	https://www.cancerrxgene.org/
	drug combination	Liu et al.^204^	http://drugcombdb.denglab.org/
	CRISPR on-target	Leenay et al.^205^	https://tdcommons.ai/single_pred_tasks/CRISPROutcome/
	CRISPR off-target	Störtz and Minary^206^	http://www.CRISPRsql.com/
	virus vector design	Bryant et al.^156^	https://www.ncbi.nlm.nih.gov/bioproject/PRJNA673640/
Clinical study (“machine learning for genomics in clinical studies”)	cross-species translation	Poussin et al.^207^	https://www.intervals.science/resources/sbv-improver/stc
	patient stratification	Curtis et al.^208^	https://www.cbioportal.org/
	patient-trial matching	Zhang et al.^182^	https://github.com/deepenroll/DeepEnroll/tree/master/Synthetic%20Data
	Mendelian randomization	Hemani et al.^189^	https://www.mrbase.org/
Post-market study (“machine learning for genomics in post-market studies”)	biomedical literature mining	Pyysalo et al.^209^	http://mars.cs.utu.fi/BioInfer/?q=download

### Genomics-related biomedical data

#### DNAs

The human genome can be thought of as the instructions for building functional individuals. DNA sequences encode these instructions. Like a computer, for which we build a program based on 0/1 bit, the basic DNA sequence units are called nucleotides (A, C, G, and T). Given a list of nucleotides, a cell can build a diverse range of functional entities (programs). There are approximately 3 billion base pairs for the human genome, and more than 99.9% are identical between individuals. If a subset of the population has different nucleotides in a genome position than the majority, this position is called a variant. This single nucleotide variant is often called a SNP. While most variants are not harmful (they are said to be functionally neutral), many correspond to the potential driver for phenotypes, including diseases.

##### Machine learning representations

**Figure 2:**
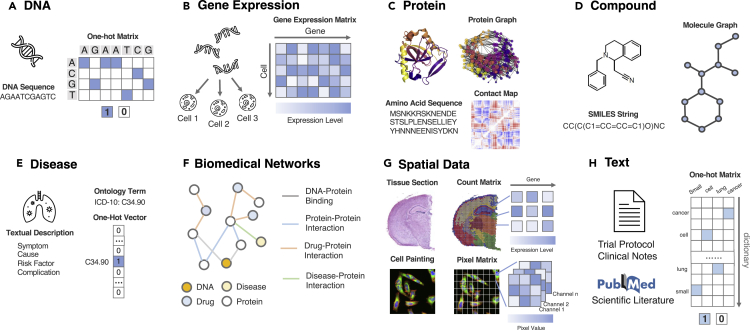
Therapeutics data modalities and their machine learning representation Detailed descriptions of each modality can be found in “genomics-related biomedical data.” (A) DNA sequences can be represented as a matrix where each position is a one-hot vector corresponding to A, C, G, T. (B) Gene expressions are a matrix of real value, where each entry is the expression level of a gene in a context such as a cell. (C) Proteins can be represented in amino acid strings, a protein graph, and a contact map where each entry is the connection between two amino acids. (D) Compounds can be represented as a molecular graph or a string of chemical tokens, which are a depth-first traversal of the graph. (E) Diseases are usually described by textual descriptions and also symbols in the disease ontology. (F) Networks connect various biomedical entities with diverse relations. They can be represented as a heterogeneous graph. (G) Spatial data are usually depicted as a 3D array, where two dimensions describe the physical position of the entity and the third dimension corresponds to colors (in cell painting) or genes (in spatial transcriptomics). (H) Texts are typically represented as a one-hot matrix where each token corresponds to its index in a static dictionary. The protein image is adapted from Gaudelet et al.;^21^ the spatial transcriptomics image is adapted from 10x Genomics; the cell painting image is from Charles River Laboratories.

A DNA sequence is a list of ACGT tokens of length *N*. It is typically represented in three ways: (1) a string ${\left\{A,C,G,T\right\}}^{N}$; (2) a two dimensional matrix $W\in R^{4\times N}$, where the *i*th column $W_{i}$ corresponds to the *i*th nucleotide and is a one-hot encoding vector of length 4, where A, C, T, and G are encoded as [1,0,0,0], [0,1,0,0], [0,0,1,0], and [0,0,0,1], respectively; or (3) a vector of ${\left\{0,1\right\}}^{N}$, where 0 means it is not a variant and 1 a variant. An example is depicted in Figure 2A.

##### Gene expression/transcripts

In a cell, the DNA sequence of each gene is transcribed into messenger RNA (mRNA) transcripts. While most cells share the same genome, the individual genes are expressed at very different levels across cells and tissue types and given different interventions and environments. These expression levels can be measured by the count of mRNA transcripts. Given a disease, we can compare the gene expression in people with the disease with expression to people in healthy cohorts (without the disease of interest) and associate various genes with the underlying biological processes in this disease. With the advance of single-cell RNA sequencing (scRNA-seq) technology, we can now obtain gene expression for the different types of cells that make up a tissue. The availability of transcripts of tens of thousands of cells creates new opportunities for understanding interactions among the behaviors of different cell types in a cell population.

##### Machine learning representations

Gene expressions/transcripts are counts of mRNA. For a scRNA-seq experiment, given *M* cells/individuals with *N* genes, we can obtain a gene expression matrix $W\in Z^{M\times N}$, where each entry $W_{i,j}$ corresponds to the transcript counts of gene *j* for cell/individual *i*. An example is depicted in Figure 2B.

#### Proteins

Most of the genes encoded in the DNA provide instructions to build a diverse set of proteins, which perform a vast array of functions. For example, transcription factors are proteins that bind to the DNA/RNA sequence and regulate their expression in different conditions. A protein is a macro-molecule and is represented by a sequence of 20 standard amino acids or residues, where each amino acid is a simple compound. Based on this sequence code, it naturally folds into a three-dimensional (3D) structure, which determines its function. As the functional units, proteins present a large class of therapeutic targets. Many drugs are designed to inhibit/promote proteins in the disease pathways. Proteins can also be used as therapeutics such as antibodies and peptides.

##### Machine learning representations

Proteins have diverse forms. For a protein with *N* amino acids, it can be represented in the following formats: (1) a string ${\left\{A,R,N,D,\ldots \right\}}^{N}$ of amino acid sequence tokens; (2) a contact map matrix $W\in R^{N\times N}$ where $W_{i,j}$ is the physical distance between *i*th and *j*th amino acids; (3) a protein graph *G* with nodes corresponding to amino acids, where nodes are connected based on rules such as a physical distance threshold or k-nearest neighbors; (4) a protein 3D grid with 3D discretized tensor, where each grid point (x,y,z) corresponds to amino acids in the 3D space. An example is depicted in Figure 2C.

#### Compounds

Compounds are molecules that are composed of atoms connected by chemical bonds. They can interact with proteins and drive important biological processes. In their natural form, compounds have a 3D structure. Small-molecule compounds are the major class of therapeutics.

##### Machine learning representations

A compound is usually represented as (1) an SMILES string where it is a depth traversal order of the molecule graph or (2) a molecular graph *G* where each node is an atom and edges are the bonds. An example is illustrated in Figure 2D.

#### Diseases

A disease is an abnormal condition that affects the function and/or modifies the structure of an organism. It is derived from factors such as genotypes, environments, and economic status, with intricate mechanisms driven by biological processes. They are observable and can be described by certain symptoms.

##### Machine learning representations

Diseases are represented by (1) symbols in the disease ontology such as ICD-10 codes or (2) text description of the specific disease. An example is depicted in Figure 2E.

#### Biomedical networks

Biological processes are not driven by individual units but consist of numerous interactions among various types of entities such as cell-signaling pathways, protein-protein interactions, and gene regulation. These interactions can be characterized by biomedical networks, where they provide a systems view toward biological phenomena. In the context of diseases, a network can also contain interactions among phenotypes or disease mechanisms. These networks are also referred to as disease maps.

##### Machine learning representations

Biomedical networks are represented as graphs, where each node is a biomedical entity and an edge corresponds to relations among them. An example is depicted in Figure 2F.

#### Spatial data

With the advance of microscopes and fluorescent probes, we can visualize cell dynamics through cellular images. By imaging cells under various conditions such as drug treatment, they allow us to identify the effect of conditions at a cellular level. Furthermore, spatial genomic sequencing techniques now allow us to visualize and understand the gene expression for cellular processes in the tissue environment.

##### Machine learning representations

Cellular image or spatial transcriptomics can be represented as a matrix of size M×N, where M,N is the width and height of the data or number of pixels/transcripts along this dimension, and each entry corresponds to the pixel of the image or the transcript count in the case of spatial transcriptomics. Additional channels (a separate matrix of size M×N) encode for information such as colors or various genes for spatial transcriptomics. After aggregation, the spatial data can be represented as a tensor of size M×N×H, where *H* is the number of channels. An example is illustrated in Figure 2G.

#### Texts

One common categorization of texts is structured versus unstructured data. Structured data follow rigid form and are easily searchable, whereas unstructured data are in a free-form format such as texts. While they are more difficult to process, they contain crucial information that usually does not exist in structured data. The first important example of text encountered in therapeutics development includes clinical trial design protocols, where texts describe inclusion and exclusion criteria for trial participation, often as a function of genome markers. For example, in a trial to study gefitinib for EGFR-mutant non-small cell lung cancer, one of the trial eligibility criteria would be “An EGFR sensitizing mutation must be detected in tumor tissue.”^22^ The second type of clinical text is clinical notes documented in EHRs. While the majority of the EHR data are structured, the unstructured clinical notes contain valuable information to support various applications such as post-market research on treatments.

##### Machine learning representations

Clinical texts are similar to texts in common natural language processing. The standard way to represent them is a matrix of size M×N, where *M* is the number of total vocabularies and *N* is the number of tokens in the texts. Each column is a one-hot encoding for the corresponding token. An example is depicted in Figure 2H.

### Machine learning methods for biomedical data

**Figure 3:**
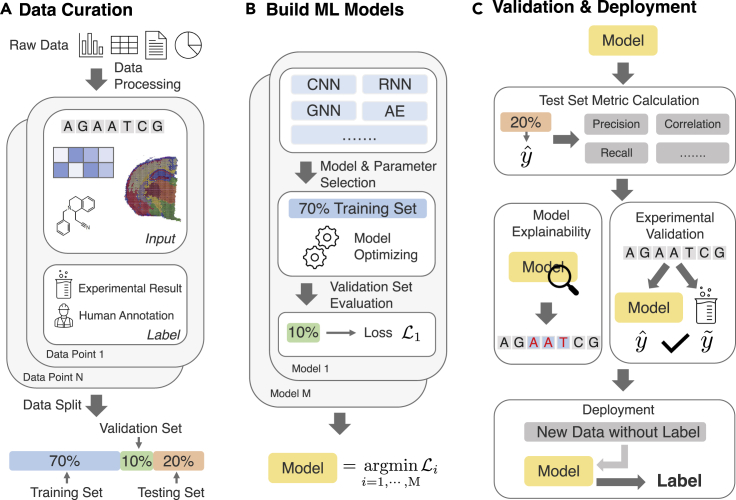
Machine learning for genomics workflow (A) The first step is to curate a machine learning dataset. Raw data are extracted from databases of various sources and are processed into data points. Each data point corresponds to an input of a series of biomedical entities and a label from annotation or experimental results. These data points constitute a dataset, and they are split into three sets. The training set is for the ML model to learn and identify useful and generalizable patterns. The validation set is for model selection and parameter tuning. The testing set is for the evaluation of the final model. The data split could be constructed in a way to reflect real-world challenges. (B) Various ML models can be trained using the training set and tuned based on a quantified metric on the validation set such as loss L that measures how good this model predicts the output given the input. Lastly, we select the optimal model given the lowest loss. (C) The optimal model can then predict on the test set, where various evaluation metrics are used to measure how good the model is on new unseen data points. Models can also be probed with explainability methods to identify biological insights captured by the model. Experimental validation is also common to ensure the model can approximate wet-lab experimental results. Finally, the model can be deployed to make predictions on new data without labels. The prediction becomes a proxy for the label from downstream tasks of interest.

ML models learn patterns from data and leverage these patterns to make accurate predictions. Numerous ML models have been proposed to tackle different challenges. This section briefly introduces the main mechanisms of popular ML models used to analyze genomic data. Figure 3 describes a typical ML for genomics data workflow. We also provide a list of public benchmarks or competitions that compare various discussed ML methods in Table 2.

**Table 2:** Public benchmarks and competitions of machine learning for therapeutics with genomics data

Name	Focus	Link
MoleculeNet	molecule learning	http://moleculenet.ai/
Therapeutics Data Commons	general therapeutics	https://tdcommons.ai/benchmark/overview/
DREAM	general biomedicine	https://dreamchallenges.org/
SBV-IMPROVER	human-mouse translation	https://www.intervals.science/resources/sbv-improver/stc
TAPE	protein engineering	https://github.com/songlab-cal/tape
CASP	protein structure	https://predictioncenter.org/
GuacaMol	molecule generation	https://www.benevolent.com/guacamol
Open Problems	single-cell analysis	https://openproblems.bio/
RxRx	cell painting	https://www.rxrx.ai/
Kaggle-MoA	mechanism of action	https://www.kaggle.com/c/lish-moa
Kaggle-HPA	single-cell classification	https://www.kaggle.com/c/hpa-single-cell-image-classification

#### Preliminary

A typical ML model for genomics usage is as follows. Given an input of a set of data points, where each data point consists of input features and a ground-truth biological label, an ML model aims to learn a mapping from input to a label based on the observed data points, which are also called training data. This setting of predicting by leveraging known supervised labels is also called supervised learning. The size of the training data is called the sample size. ML models are data-hungry and usually need a large sample size to perform well.

The input features can be DNA sequences, compound graphs, or clinical texts, depending on the task at hand. The ground-truth label is usually obtained via biological experiments. The ground truth also presents the goal for an ML model to achieve. Thus, if the ground-truth label contains errors (e.g., human labeling error or wet-lab experiments error), the ML model could optimize over the wrong signals, highlighting the necessity of high-quality data curation and control. It is also worth mentioning that the input can also present quality issues, such as shifts of the cell image, batch effect for gene expressions, and measurement errors. There are various forms of ground-truth labels. If the labels are continuous (e.g., binding scores), the learning problem is a regression problem. And if the labels are discrete variables (e.g., the occurrence of interaction), the problem is a classification problem. Models focusing on predicting the labels of the data are called discriminative models. Besides making predictions, ML models can also generate new data points by modeling the statistical distribution of data samples. Models following this procedure are called generative models.

When labels are not available, an ML model can still identify the underlying patterns within the unlabeled data points. This problem setting is called unsupervised learning, whereby models discover patterns or clusters (e.g., cell types) by modeling the relations among data points. Self-supervised learning uses supervised learning methods for handling unlabeled data. It creatively produces labels from the unlabeled data (e.g., masking out a motif and using the surrounding context to predict the motif).^14^^,^^23^

In many biological cases ground-truth labels are scarce, in which case few-shot learning can be considered. Few-shot learning assumes only a few labeled data points but many unlabeled data points. Another strategy is called meta-learning, which aims to learn from a set of related tasks to form the ability to learn quickly and accurately on an unseen task.

If a model integrates multiple data modalities (e.g., DNA sequence plus compound structure), it is called multi-modal learning. When a model predicts multiple labels (e.g., multiple target endpoints), it is called multi-task learning.

In biomedical ML problems, high-quality data curation is a key step. Biomedical data are usually generated from wet-lab experiments, and are thus prone to numerous experimental glitches. A dataset usually results from different biotechnology platforms, batches, time points, and conditions. Thus, accurate and careful pre-processing and data fusion are tremendously important; otherwise, the ML model may learn from biased or erroneous data. Numerous data-processing protocols have also been formulated, such as batch-effect corrections,^24^ imputation,^25^ and datasets integration.^26^ Efforts in curating ML-ready therapeutics datasets have also been initiated.^27^

In this survey, we argue that the integration of genomics data with other biomedical entity types is the key to transforming data into therapeutic products. This integration also goes beyond integrating different contexts such as cellular, tissue, and organism at temporal scales. Data integration in ML is a well-studied subject. There are mainly three categories.^28^ The first is at the dataset level, where datasets are fused and aligned to form a combined dataset and then fed into the ML model. The second is for each data type or dataset foe which a separate ML model is used to encode it, after which the ML models are combined in the ML latent space. The third is integration on the output space, where model predictions are aggregated through ensembles. For each task covered in this survey, we specify the data-integration strategy.

#### Classic ML models

**Figure 4:**
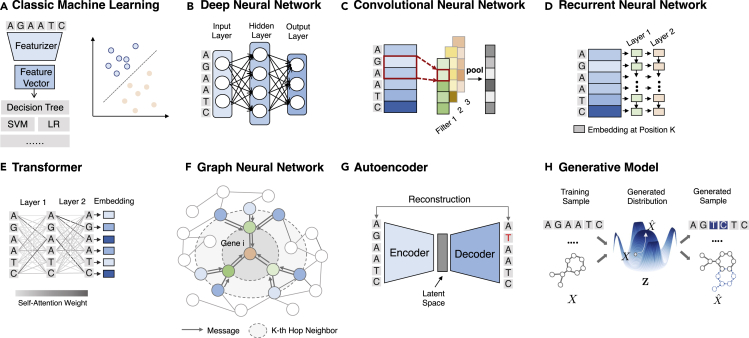
Illustrations of machine learning models Details about each model can be found in “machine learning methods for biomedical data.” (A) Classic machine learning models featurize raw data and apply various models (mostly linear) to classify (e.g., binary output) or regress (e.g., real value output). (B) Deep neural networks map input features to embeddings through a stack of non-linear weight multiplication layers. (C) Convolutional neural networks apply many local filters to extract local patterns and aggregate local signals through pooling. (D) Recurrent neural networks generate embeddings for each token in the sequence based on the previous tokens. (E) Transformers apply a stack of self-attention layers that assign a weight for each pair of input tokens. (F) Graph neural networks aggregate information from the local neighborhood to update the node embedding. (G) Autoencoders reconstruct the input from an encoded compact latent space. (H) Generative models generate novel biomedical entities with more desirable properties.

Traditional ML usually requires a transformation of input to tabular real-valued data, where each data point corresponds to a feature vector. In our context, these are pre-defined features such as the SNP vector, polygenic risk scores, and chemical fingerprints. These tabular data can then be fed into a wide range of supervised models, such as linear/logistic regression, decision trees, random forest, support vector machine, and naive Bayes.^29^ They work well when the features are well defined. A multi-layer perceptron^30^ (MLP) consists of at least three layers of neurons, where each layer is fed into a non-linear activation function to capture these patterns. When the number of layers is large, it is called a deep neural network (DNN). Classic ML models are very simple to implement and are highly scalable. They can serve as a strong baseline. However, they only accept real-valued vectors as inputs and do not fit the diverse biomedical entity types such as sequence and graph. Also, these vectors are usually features engineered by humans, which further limits their predictive powers. Examples are shown in Figures 4A and 4B.

##### Suitable biomedical data

Any real-valued feature vectors built upon biomedical entities such as SNP profile and chemical fingerprints.

#### Convolution neural network

Convolution neural networks (CNNs) represent a class of DNNs widely applied for image classification, natural language processing, and signal processing such as speech recognition.^31^ A CNN model has a series of convolution filters, which allow it to identify local patterns in the data (e.g., edges, shapes for images). Such networks can automatically extract hierarchical patterns in data. The weight of each filter reveals patterns. CNNs are powerful in picking up the patterns locally, which is ideal for biomedical tasks whereby local structures are important to the outcome, such as consecutive blocks of genes (conserved motifs) in DNA sequence and substructures in compound string representation. However, they are restricted to grid-structured data and do not work for non-Euclidean objects such as gene regulatory networks or 3D biochemical structures. Also, CNNs are translation invariant, which is a double-edged sword. On the one hand, it can robustly predict even if one perturbs the input by translation. On the other hand, it might not be ideal with biomedical data whereby order/spatial location information is crucial for the outcome such as time-series gene expression trajectory. An example is depicted in Figure 4C.

##### Suitable biomedical data

Short DNA sequence, compound SMILES strings, gene expression profile, and cellular images.

#### Recurrent neural network

A recurrent neural network (RNN) is designed to model sequential data, such as time series, event sequences, and natural language text.^32^ The RNN model is sequentially applied to a sequence. The input at each step includes the current observation and the previous hidden state. RNN is natural to model variable-length sequences. There are two widely used variants of RNNs: long short-term memory (LSTM)^33^ and gated recurrent units.^34^ RNNs are natural candidates for sequential data such as DNA/protein sequence and textual data, where the next token depends on previous tokens. However, they often suffer from the vanishing gradient problem, which precludes them from modeling long-range sequences. Thus, it is not ideal for a long DNA sequence. An example is depicted in Figure 4D.

##### Suitable biomedical data

DNA sequence, protein sequence, and texts.

#### Transformer

Transformers^35^ are a recent class of neural networks that leverage self-attention: assigning a score of interaction among every pair of input features (e.g., a pair of DNA nucleotides). By stacking these self-attention units, the model can capture more expressive and complicated interactions. Transformers have shown superior performances on sequence data, such as natural language processing. They have also been successfully adapted for state-of-the-art performances on proteins^36^ and compounds.^37^ Transformers are powerful, but they are not scalable due to the expensive self-attention calculation. Despite several recent advances to increase the maximum size to the order of tens of thousands,^38^ this limitation has still prevented its usage for extremely long sequences such as genome sequences and usually requires partitioning and aggregation strategies. An example is depicted in Figure 4E.

##### Suitable biomedical data

DNA sequence, protein sequence, texts, and image.

#### Graph neural networks

Graphs are universal representations of complex relations in many real-world objects. In biomedicine, graphs can represent knowledge graphs, gene expression similarity networks, molecules, protein-protein interaction networks, and medical ontologies. However, graphs do not follow rigid data structures as in sequences and images. Graph neural networks (GNNs) are a class of model that converts graph structures into embedding vectors (i.e., node representation or graph representation vectors).^39^ In particular, GNNs generalize the concept of convolution operations to graphs by iterative passing and aggregating messages from neighboring nodes. The resulting embedding vectors capture the node attributes and the network structures. GNNs are a powerful tool to model any graph-structured biomedical data. However, when adapting GNNs to the biomedical domain, special attention is required. For example, GNNs heavily rely on the assumption of homophily, where similar nodes are connected. In biomedical networks, however, it has been shown to exhibit more complicated behavior such as skip similarity.^40^ Besides, the local message-passing schemes oversimplify biochemical graph structures. Domain-motivated GNN design where biophysical principles are integrated is highly desirable.^41^ An example is depicted in Figure 4F.

##### Suitable biomedical data

Biomedical networks, compound/protein graphs, and similarity network.

#### Autoencoders

Autoencoders (AEs) are an unsupervised method in deep learning. AEs map the input data into a latent embedding (encoder) and then reconstruct the input from the latent embedding (decoder).^42^ Their objective is to reconstruct the input from a low-dimensional latent space, thus allowing the latent representation to focus on essential properties of the data. Both encoders and decoders are neural networks. AEs can be considered as a non-linear analog to principal component analysis. The generated latent representation captures patterns in the input data and can thus be used to carry out unsupervised learning tasks such as clustering. Among its variants, the denoising autoencoders take partially corrupted inputs and are trained to recover original undistorted inputs.^43^ Variational autoencoders (VAEs) model the latent space with probabilistic models. As these probabilities are complex and usually intractable, they adopt a variational inference technique to approximate these probabilistic models.^44^ AEs are widely used to map gene expression to latent states without any labels, and these latent embeddings are useful for downstream single-cell analyses. One disadvantage of AEs is that they model training data, while in single-cell analysis test data can come from different settings from training data. It is thus challenging to obtain accurate latent embeddings with AEs on novel test data. An example is depicted in Figure 4G.

##### Suitable biomedical data

Unlabeled data.

#### Generative models

In contrast to making a prediction, generative models aim to learn a sufficient statistical distribution that characterizes the underlying datasets (e.g., a set of DNA sequences for a disease) and its generation process.^45^ Based on the learned distribution, various kinds of downstream tasks can be supported. For example, from this distribution one can intelligently generate optimized data points. These optimized samples can be novel images, compounds, or RNA sequences. One popular model is called generative adversarial networks (GANs)^46^ consisting of two submodels: a generator that captures the data distribution of a training dataset in a latent representation and a discriminator that determines whether a sample is real or generated. These two submodels are trained iteratively such that the resulting generator can produce realistic samples that potentially fool the discriminator. An example is depicted in Figure 4H.

##### Suitable biomedical data

Data in which new variants can have more desirable properties (e.g., molecule generation for drug discovery).^47^^,^^48^ Depending on the data modality, different encoders can be chosen for the generative models.

## Machine learning for genomics in target discovery

A therapeutic target is a molecule (e.g., a protein) that plays a role in the disease's biological process. The molecule could be targeted by a drug to produce a therapeutic effect such as inhibition, thereby blocking the disease process. Much of target discovery relies on fundamental biological research in depicting a full picture of human biology and, based on this knowledge, to identify target biomarkers. In this section, we review ML for genomics tasks in target discovery. First, we review six tasks that use ML to facilitate understanding of human biology, and second, we describe four tasks in using ML to help identify druggable biomarkers more accurately and more quickly.

### Facilitating understanding of human biology

Oftentimes, the first step for developing any therapeutic agent is to generate a disease hypothesis and understand the disease mechanisms. This requires some understanding of basic human biology, since diseases are complicated and driven by many factors. ML applied to genomics can facilitate basic biomedical research and help us to understand disease mechanisms. A wide range of relevant tasks have been tackled by ML, from predicting splicing patterns,^49^^,^^50^ DNA methylation status,^51^ to decoding the regulatory roles of genes.^52^^,^^53^ The majority of previous reviews have focused on this theme only. Many tasks could be covered in this theme. In this review, we chose six important tasks based on the following criteria: (1) the task is closely tight to understanding disease mechanism and discovering targets, as this survey focuses on therapeutics development; (2) the task is popular (i.e., sufficient literature exists), and ML has successfully been applied to it; (3) the overall selection is diverse to cover different data modalities, ML task formulation and ML representations (e.g., graphs, images, vectors). For a full review of biological understanding, we refer readers to Angermueller et al.^54^

#### DNA-protein and RNA-protein binding prediction

DNA-binding proteins bind to specific DNA strands (binding sites/motifs) to influence the transcription rate to RNA, chromatin accessibility, and so forth. These motifs regulate gene expression and, if mutated, can potentially contribute to diseases. Similarly, RNA-binding proteins bind to RNA strands to influence RNA processing, such as splicing and folding. Thus, it is important to identify the DNA and RNA motifs for these binding proteins.

Traditional approaches are based on position weight matrices (PWMs), but they require existing knowledge about the motif length and typically ignore interactions among the binding-site loci. ML models trained directly on sequences to predict binding scores circumvent these challenges. A CNN is a great match for this task because CNN's filters operate in a mechanism similar to that of PWMs by convolving over snippets of motifs and assigning higher weights to the motifs that are important. We can also examine binding-site motifs through visualizing CNN filter weights. Based on this key observation, various methods have been proposed. For example, Alipanahi et al.^55^ use a CNN to train large-scale DNA/RNA sequences with varying lengths to predict the binding scores. While DNA sequence alone provides strong signals, other channels of information could further aid the binding prediction. For example, Kircher et al.^56^ show that including evolutionary features for identifying chromatin proteins/histone marks binding can further improve the performance. Similarly, Zhou and Troyanskaya^53^ show that integrating another CNN model on additional information from the epigenomics profile further improves performance. Extending CNN-based models, a large body of works has been proposed to predict DNA- and RNA-protein binding.^57^^,^^58^, ^59^, ^60^ While CNN models are highly predictive, the interpretability is limited in its resolution, as the CNN filter has a window size of around 100–200 base pairs. The base-resolution model is highly ideal for identifying granular information such as transcription factor (TF) cooperativity. Recently, Avsec et al.^61^ have shown the benefits of the base-resolution CNN model in TF binding prediction.

##### Machine learning formulation

**Figure 5:**
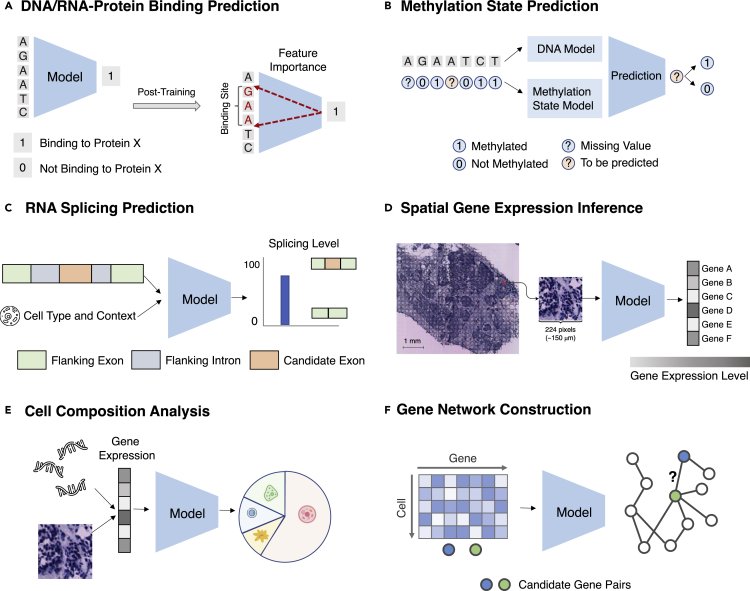
Task illustrations for the theme “facilitating understanding of human biology” (A) A model predicts whether a DNA/RNA sequence can bind to a protein. After training, one can identify binding sites based on feature importance (see “DNA-protein and RNA-protein binding prediction”). (B) A model predicts missing DNA methylation state based on its neighboring states and DNA sequence (see “methylation state prediction”). (C) A model predicts the splicing level given the RNA sequence and the context (see “RNA splicing prediction”). (D) A model predicts spatial transcriptomics from tissue image (see “spatial gene expression inference”). (E) A model predicts the cell-type compositions from the gene expression (see “cell-composition analysis”). (F) A model constructs a gene regulatory network from gene expressions (see “gene network construction”. Panel (C) is adapted from Xiong et al.,^50^ and the spatial transcriptomics image in panel (D) is from Bergenstråhle et al.^62^

Given a set of DNA/RNA sequences, predict their binding scores. After training, use feature importance attribution methods to identify the motifs. An illustration of the task is presented in Figure 5A.

#### Methylation state prediction

DNA methylation adds methyl groups to individual A or C bases in the DNA to modify gene activity without changing the sequence. It is a commonly used mediator for biological processes such as cancer progression and cell differentiation.^63^ Thus, it is important to know the methylation status of DNA sequences in various cells. However, since the single-cell methylation technique has low coverage, most of the methylation status at specific DNA positions is missing, requiring accurate imputation.

Classical methods can only predict population-level status given features instead of cell-level status because cell-level prediction requires granular and complex modeling of long sequential methylation status.^64^^,^^65^ Sequential ML models such as RNNs and CNNs are ideal choices because they can capture the non-linear sequential dependencies that are hidden in the methylation sequence. For example, given a set of cells with their available sequenced methylation status for each DNA position and the DNA sequence, Angermueller et al.^51^ accurately infer the unmeasured methylation statuses at a single-cell level. More specifically, the imputation of DNA methylation positions uses a bidirectional RNN on a sequence of cells' neighboring available methylation states and a CNN on the DNA sequence. The combined embedding takes into account information between DNA and methylation status across cells and within cells. Alternative architecture choices have also been proposed, such as using Bayesian clustering^66^ or a variational AE.^67^ Notably, it can also be extended to RNA methylation state prediction. Zou et al.^68^ apply CNN on the neighboring methylation status and the word2vec model on RNA subsequence for RNA methylation status prediction. The main challenge in DNA methylation prediction is the number of CpG sites, which could be many millions. The ability of the model to accommodate such long-range information is limited in current ML models due to issues such as vanishing gradient problems in RNN and scalability issues for transformers.

##### Machine learning formulation

For a DNA/RNA position with missing methylation status, given its available neighboring methylation states and the DNA/RNA sequence, predict the methylation status on the position of interest. The task is illustrated in Figure 5B.

#### RNA splicing prediction

RNA splicing is a mechanism to assemble the coding regions and remove the non-coding ones to be translated into proteins. A single gene can have various functions by splicing the same gene in different ways given different conditions. López-Bigas et al.^69^ estimate that as many as 60% of pathogenic variants responsible for genetic diseases may influence splicing. Gefman et al.^70^ identify around 2% of synonymous variants and 0.6% of intronic variants as likely pathogenic due to alternative splicing defects. Thus, it is important to be able to identify the genetic variants that cause alternative splicing. Traditional wet-lab measurements of splicing levels are highly unscalable.

Xiong et al.^50^ pioneer the use of ML in splicing prediction. They model this problem as predicting the splicing level of an exon, measured by the transcript counts of this exon, given its neighboring RNA sequence and the cell-type information. It uses Bayesian neural network ensembles on top of curated RNA features and has demonstrated its accuracy by identifying known mutations and discovering new ones. Notably, this model is trained on large-scale data across diverse disease areas and tissue types. Thus, the resulting model can predict the effect of a new unseen mutation within hundreds of nucleotides on the splicing of an intron without experimental data. This property of generalization across new contexts is crucial but is particularly difficult for ML due to its natural tendency to overfit on spurious correlation. In addition, to predict the splicing level given a triplet of exons in various conditions, recent models have been developed to annotate the nucleotide branchpoint of RNA splicing. Paggi and Beherano^71^ feed an RNA sequence into an RNN, predicting the likelihood of being a branchpoint for each nucleotide. Jagadeesh et al.^72^ further improve the performance by integrating features from the splicing literature and generate a highly accurate splicing-pathogenicity score.

##### Machine learning formulation

Given an RNA sequence and its cell type, if available, for each nucleotide, predicts the probability of being a spliced breakpoint and the splicing level. The task is illustrated in Figure 5C.

#### Spatial gene expression inference

Gene expression varies across the spatial organization of tissue. This heterogeneity contains important insights into the biological effects. Regular sequencing, whether of single cells or bulk tissue, does not capture this information. Recent advances in spatial transcriptomics characterize gene expression profiles in their spatial tissue context.^73^ However, integrating the sequencing output with the tissue context provided by histopathology images takes resources and time. Automatic annotations could drastically save resources. He et al.^62^ introduce ML to this important problem by formulating it as gene expression prediction from histopathology images. As a histopathology slide is an image, a natural model is through CNN. They develop a deep CNN that predicts gene expression from histopathology of patients with breast cancer at a resolution of 100 μm. They also show that the model can generalize to other breast cancer datasets without retraining. Building upon the inferred spatial gene expression levels, many downstream tasks are enabled. For example, Levy-Jurgenson et al.^74^ construct a pipeline that characterizes tumor heterogeneity on top of the CNN gene expression inference step. Bergenstråhle et al.^75^ model the spatial transcriptomics and histology image jointly through latent state and infer high-resolution denoised gene expression by posterior estimation. Despite the promises, one crucial challenge of this task is the requirement of a large number of ground-truth annotations, which are expensive to acquire in a novel set of histopathology slides or genes. This challenge highlights the need for the model to learn from few examples by adopting techniques such as meta-learning or transfer learning.

##### Machine learning formulation

Given the histopathology image of the tissue, predict the gene expression for every gene at each spatial transcriptomics spot. The task is illustrated in Figure 5D.

#### Cell-composition analysis

Different cell types can drive changes in gene expressions that are unrelated to the interventions. Analyzing the average gene expression for a batch of mixed cells with distinct cell types could lead to bias and false results.^76^ Thus, it is important to deconvolve the gene expressions of the cell-type composition from the real signals for tissue-based RNA-seq data.

ML models can help estimate the cell-type proportions and the gene expression. The rationale is to obtain parameters of gene expression (a signature matrix) that characterize each cell type through single-cell profiles. The signature matrix should contain gene expressions that are stably expressed across conditions. These parameters are then integrated into the RNA-seq data to infer cell composition for a set of query gene expression profiles. Various methods, including linear regression^77^ and support vector machines,^78^ are used to predict a cell-composition vector when combined with the signature matrix to approximate the gene expression. In these works the signature matrix is pre-defined, which may not be optimal. A learnable signature matrix could lead to improved accuracy. Pioneering this direction, Menden et al.^79^ apply DNNs to predict cell-composition profile directly from the gene expression, where the hidden neurons can be considered as the learned signature matrix. Cell deconvolution is also crucial for spatial transcriptomes where each spot could contain 2 to 20 cells from a mixture of dozens of possible cell types. Andersson et al.^80^ model various cell-type-specific parameters using a customized probabilistic model. As spots in a slide have spatial dependencies, modeling them as a graph can further improve performance. Notably, Su and Song^81^ initiate the use of graph convolutional network to leverage information from similar spots in the spatial transcriptomics. There are two major challenges for this task. The first is the quality of the gold-standard annotations as the cell-proportion estimates are usually noisy. This calls for ML methods that can model the label noise.^82^ Another challenge is that the proportions are highly dependent on phenotypes such as age, gender, and disease status. How to take into account this information in the ML models is also valuable for more accurate deconvolution.

##### Machine learning formulation

Given the gene expressions of a set of cells (in bulk RNA-seq or a spot in spatial transcriptomics), infer proportion estimates of each cell type for this set. The task is illustrated in Figure 5E.

#### Gene network construction

The expression levels of a gene are regulated via TFs produced by other genes. Aggregating these TF-gene relations results in the gene regulatory network. Accurate characterization of this network is crucial because it describes how a cell functions. However, it is difficult to quantify gene networks on a large scale through experiments alone.

Computational approaches have been proposed to construct gene networks from gene expression data. The majority of them learn a mapping from expressions of a gene to TF. If the mapping is successful, it is likely that this TF affects this gene. Various mapping methods using classic ML have been proposed, such as linear regression,^83^ random forest,^84^ and gradient boosting.^85^ However, gene networks constructed through these methods are not controllable in sparsity and are sensitive to parameter changes, and thus are filled with noises. Recently, Shrivastava et al.^86^ introduced a specialized unrolled algorithm to control the sparsity of the learned network. They also leveraged supervision obtained through synthetic data simulators to improve robustness further. Despite the promises, gene network construction is difficult due to the sparsity, heterogeneity, and noise of the gene expression data, particularly the diverse datasets from the integration of scRNA-seq experiments. The clinical validation of the predicted gene associations also poses challenges, since it is difficult to screen such a large set of predictions.

##### Machine learning formulation

Given a set of gene expression profiles of a gene set, identify the gene regulatory network by predicting all pairs of interacting genes. The task is illustrated in Figure 5F.

### Identifying druggable biomarkers

Diseases are driven by complicated biological processes in which each step may be associated with a biomarker. By identifying these biomarkers, we can design therapeutics to break the disease pathway and cure the disease. Machine learning can help identify these biomarkers by mining through large-scale biomedical data to predict genotype-phenotype associations accurately. Probing the trained models can uncover potential biomarkers and identify patterns related to the disease mechanisms. Next, we present several important tasks related to biomarker identification.

#### Variant calling

Variant calling is the very first step before relating genotypes to diseases. It is used to specify which genetic variants are present in each individual's genome from sequencing. The majority of the variants are biallelic, meaning that each locus has only one possible alternative form of nucleotide compared with the reference, while a small fraction is also multi-allelic, meaning that each locus can have more than one alternative form. As each locus has two copies, one from mother and another from father, the variant is measured by the total set of nucleotides (e.g., for biallelic variant, suppose B is the reference nucleotide and b is the alternative; three genotypes are possible: homozygous [BB], heterozygous [Bb], and homozygous alternate [bb]). Raw sequencing outputs are usually billions of short reads, and these reads are aligned to a reference genome. In other words, for each locus we have a set of short reads that contain this locus. Since sequencing techniques have errors, the challenge is to predict the variant status of this locus accurately from the set of reads. Manual processing of such a large number of reads to identify each variant is infeasible. Thus, efficient computational approaches are needed for this task.

A statistical framework called the Genome Analysis Toolkit (GATK),^87^ which combines logistic regression, hidden Markov models, and Gaussian mixture models, is commonly used for variant calling. While previous works operate on sequencing statistics, DeepVariant^88^ treats the sequencing alignments as images. The images are raw data and they contain more information than the engineered sequencing features. It then applies CNN to extract useful signals and has been shown to have superior performance to previous modeling efforts. DeepVariant also works for multi-allelic variant calling. In addition to predicting zygosity, Luo et al.^89^ use multi-task CNNs to predict the variant type, alternative allele, and indel length. Many other deep learning-based methods are proposed to tackle more specific challenges, such as long sequencing length using LSTMs.^90^ Benchmarking efforts have also been conducted.^91^ Although most methods have greater than 99% accuracy, thousands of variants are still being called incorrectly, since the genome sequence is extremely long. How to adjust ML models to focus on the hard locus is a promising direction. Besides, variability persists across different sequencing technologies. Another challenge is the phasing problem, which estimates whether the two mutations in a gene are on the same chromosome (haplotypes) or opposite ones.^92^ Recently, Zhao et al.^93^ have extended the prediction of variants from RNA-seq gene expression data with improved accuracy over DNA-based data. This suggests another potentially promising avenue for future research or a complementary approach with DNA-based methods to reduce misclassification.^94^

##### Machine learning formulation

**Figure 6:**
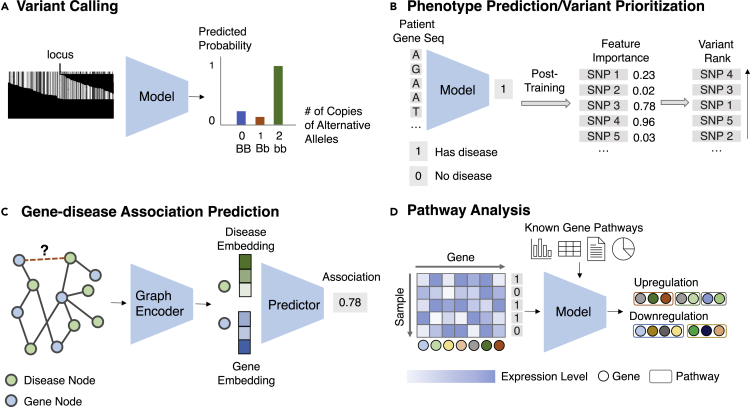
Task illustrations for the theme “identifying druggable biomarkers” (A) A model predicts the zygosity given a read pileup image (see “variant calling”). (B) A model predicts whether the patient is at risk for the disease given the genomic sequence. After training, feature importance attribution methods assign importance for each variant, which is then ranked and prioritized (see “variant pathogenicity prioritization/phenotype prediction”). (C) A graph encoder obtains embeddings for each disease and gene node, and they are fed into a predictor to predict their association (see “gene-disease association prediction”). (D) A model identifies a set of gene pathways from the gene expression profiles and the known gene pathways (see “pathway analysis and prediction”).

Given the aligned sequencing data ([1] read pileup image, which is a matrix of dimension *M* and *N*, with *M* the number of reads and *N* the length of reads; or [2] the raw reads, which are a set of sequences strings) for each locus, classify the multi-class variant status. The task is illustrated in Figure 6A.

#### Variant pathogenicity prioritization/phenotype prediction

There are many genomic variants in the human genome, at least 1 million per person. While many influence complex traits and are relatively harmless, some are associated with diseases. Complex diseases are associated with multiple variants in both coding and non-coding regions of the genome. Thus, prioritization of pathogenic variants from the entire variant set can potentially lead to disease targets.

There are mainly two computational approaches. The first one is to predict the pathogenicity given a set of features for a single variant. These features are usually curated from biochemical knowledge, such as amino acid identities. Kircher et al.^56^ build on these features using a linear support vector machine and Quang et al.^95^ use deep neural networks to classify whether a variant is pathogenic. DNN shows improved performance on classification metrics. After training, the model can generate a ranked list of variants based on their predicted pathogenicity likelihood whereby the top ones are prioritized. Note that this line of work considers each variant as an input data point and assumes some knowledge of the pathogenicity of the variants, which is not the case in many scenarios, especially for new diseases.

Another line of work is to use each genome profile as a data point and use a computational model to predict disease risks from this profile. If the model is accurate, one can obtain variants contributing to the prediction of the disease phenotype. Predicting directly from the whole-genome sequence is challenging for two reasons. First, as the whole-genome is high-dimensional while the cohort size for each disease is relatively limited, this presents the “curse of dimensionality” challenge in ML. Second, most SNPs in the input genome are irrelevant to the disease, presenting difficulty in correctly identifying these signals from the noise. Kooperberg et al.^96^ use a sparse regression model to predict the risk of Crohn's disease for patients using genomics data in the coding region. Paré et al.^97^ use gradient boosted regression to approximate polygenic risk score for complex traits such as diabetes, height, and body mass index. Isgut et al.^98^ use logistic regression on polygenic risk scores to improve myocardial infarction risk prediction. Zhou et al.^99^ apply DNNs on the epigenomic features of both the coding and non-coding regions to predict gene expression for more than 200 tissue and cell types and later identify disease-causing SNPs. Building upon DeepSEA, Zhou and colleagues^53^^,^^100^ apply CNN on epigenomic profiles, which are modifications of the DNA sequence such as DNA methylation or chromatin accessibility, to predict autism and identify experimentally validated non-coding variant mutations. ML models usually output multiple potential candidates of biomarkers, each associated with a value estimating the likelihood for being pathogenic. The standard procedure includes a post-training ranking step to retrieve the top-K biomarkers based on the pathogenic likelihood. However, in many cases, the model ends up with many potential candidates, limiting their utility. To circumvent this issue, sparsification of the model might be useful by tricks such as adding L1 penalization of the model output and model pruning.^101^ Besides, injecting model uncertainty score could be leveraged to better inform the prediction by removing scores with low certainty.^102^

##### Machine learning formulation

Given features about a variant, predict its corresponding disease risk and then rank all variants based on the disease risk. Alternatively, given the DNA sequence or other related genomics features, predict the likelihood of disease risk for this sequence and retrieve the variant in the sequence that contributes highly to the risk prediction. The task is illustrated in Figure 6B.

#### Rare disease detection

In the United States, a rare disease is defined as one that affects fewer than 200,000 people, with other countries similarly defining a rare disease based on low prevalence. There are around 7,000 rare diseases, which collectively affect 350 million people worldwide.^103^ Due to limited financial incentives, unknown disease mechanisms, and potential difficulties in recruiting sufficient patients for clinical trials, more than 90% of rare diseases lack effective treatments. Also, initial misdiagnosis is common. On average, it takes more than 7 years and eight physicians for a patient to be correctly diagnosed. Importantly, it is likely that targets identified for rare diseases may also be useful for therapeutic intervention of similar more common diseases.

ML models are good at identifying patterns from complex patient data. Rare disease detection can be formulated as a classification task, similar to phenotype prediction. It aims to identify whether the patient has a rare disease from the patient's genomic sequence and information such as EHRs. If sufficient data from patients with a rare disease and suitable controls exist, ML models can be applied to detect rare diseases. Also, the genetic complexity of rare diseases is that they have missing heritability, which could be harbored in regulatory regions instead of the coding regions. Leveraging this important knowledge, Yin et al.^104^ propose a two-step CNN approach whereby one CNN first predicts the promoter regions likely associated with amyotrophic lateral sclerosis. Another CNN detects whether the patient has a rare disease based on genotypes in the selected genomic regions.

However, rare diseases pose special challenges to ML compared with classical phenotype prediction because these diseases have an extremely low prevalence in the data while most data points belong to the control set. This data imbalance makes it difficult for ML models to pick up signals and prevent them from making an accurate prediction. Thus, special model designs are required. The standard way for data imbalance includes oversampling the rare cases or downsampling the majority of cases. A more sophisticated and powerful strategy is using synthetic data by generating fake but realistic rare cases. Popular approaches combine minority points to forge a new point.^105^, ^106^, ^107^ Intelligent synthetic data generation by modeling the minority data distribution through generative models could lead to more realistic samples. Cui et al.^108^ pioneer a generative adversarial network (GAN) model to generate synthetic but realistic rare disease patient embeddings to alleviate the class imbalance problem and show a significant performance increase in rare disease detection. Besides generating realistic data, low-resource learning techniques can also be applied to rare disease cases. For example, Taroni et al.^109^ use a transfer learning framework to adapt a smaller set of rare disease genomic data from large-scale genomic data with a diverse set of diseases. Specifically, they leverage biological principles by constructing latent variables shared across a wide range of diseases. These variables correspond to genetic pathways. As these variables are the fundamental biology units, they can be naturally adopted even for smaller datasets such as rare disease cohorts.

##### Machine learning formulation

Given the gene expression data and other auxiliary data of a patient, predict whether this patient has a rare disease. Also, identify genetic variants for this rare disease. The task is illustrated in Figure 6B, which is the same as phenotype prediction.

#### Gene-disease association prediction

Although numerous genes are now mapped to diseases, human knowledge about gene-disease association mapping is vastly incomplete. At the same time, we know many genes are similar to each other, as is also the case for diseases. We can impute unknown associations from known ones by many similarity rules that govern the gene-disease networks to leverage these similarities. One notable rule is the “guilt by association” principle.^110^ For example, disease *X* and gene *a* are more likely to be associated if we know gene *b* associated with disease *X* has a similar functional role as gene *a*. In contrast to variant prioritization focusing on predicting one specific disease, gene-disease association predictions aim to predict any disease-gene pairs.

Many graph-theoretic approaches such as diffusion^111^ have been applied to gene-disease association prediction. However, they require strong assumptions about the data. Learnable methods have also been heavily investigated. Studies have shown that integrating similarity across multiple data types can help gene-disease prediction.^112^ Notably, Luo et al.^113^ fuse information from protein-protein interaction and gene ontology through a multi-modal deep belief network. Cáceres and Paccanaro^114^ use phenotype data to transfer knowledge from other phenotypically similar diseases using a network diffusion method, whereby the phenotypical similarity is defined by the distance on the disease ontology trees. As gene-disease relations can be viewed as graphs, GNN is an ideal modeling choice by formulating it as a link prediction problem. However, GNNs highly rely on the principle of homophily in social networks whereas biomedical interaction networks present more complicated graph connectivity. Notably, Huang et al.^40^ observe the skip similarity in biomedical graphs and propose a novel GNN to improve gene-disease association prediction. As some diseases such as rare diseases are not well annotated compared with other common diseases, predicting molecularly uncharacterized (no known biological function or genes) diseases is difficult but crucial. This poses a special requirement for ML models to generalize to low-represented data groups, often formulated under the long-tail prediction regime.^115^

##### Machine learning formulation

Given the known gene-disease association network and auxiliary information, predict the association likelihood for every unknown gene-disease pair. The task is illustrated in Figure 6C.

#### Pathway analysis and prediction

Many diseases are driven by a set of genes forming disease pathways. Pathway analysis identifies these gene sets through transcriptomics data and leads toward a more complete understanding of disease mechanisms. Many statistical approaches have been proposed. For example, Gene Set Enrichment Analysis^116^ leverages existing known pathways and calculates statistics on omics data to see whether any pathway is activated. However, it treats each pathway as a set while no relation among the genes is provided. Other topology-based pathway analyses^117^ that take into account the gene relational graph structure are also proposed. Many pathway analyses suffer from noise and provide unstable pathway activation and inhibition patterns across samples and experiments. Ozerov et al.^118^ introduce a clustered gene importance factor to reduce noise and improve robustness. Current pathway analysis heavily relies on network-based methods.^119^ Another approach is to understand potential disease mechanisms by probing explainable ML models that predict genotype-to-disease association. Explainable artificial intelligence (AI) models identify small gene sets or a gene subgraph that mostly contribute to the prediction. However, this requires modeling the underlying biological processes. Many efforts have been made to simulate cell-signaling pathways and corresponding hierarchical biological processes *in silico*. Karr et al.^120^ devised the first whole-cell approach to predict cell growth from genotype using a set of differential equations. Recently, an ML model called visible neural network^121^ simulates the hierarchical biological processes (gene ontology) in a eukaryotic cell as a feedforward neural network where each neuron corresponds to a biological subsystem. This model is trained end-to-end from genotype to cell fitness phenotype with good accuracy. A post hoc interpretability method that assigns scores for each subsystem generates a likely mechanism for the fitness of a cell after training. This method has been extended recently to train on genomics data related to prostate cancer phenotype to generate disease pathways.^122^

##### Machine learning formulation

Given the gene expression data for a phenotype and known gene relations, identify a set of genes corresponding to disease pathways. The task is illustrated in Figure 6D.

## Machine learning for genomics in therapeutics discovery

After a drug target is identified, a campaign to design potent therapeutic agents to modulate the target and block the disease pathway is initiated. These therapeutics can be a small molecule, an antibody, or gene therapy, among others. The discovery consists of numerous phases and subtasks to ensure the efficacy and safety of the therapeutics. Genomics data also play a role in this process. In this section, we review ML for genomics in therapeutics discovery under two main themes. We first investigate the relation of small-molecule drug efficacy given different cellular genomic contexts. We then review how ML can enable the design of various gene therapies.

### Improving context-specific drug response

Precision medicine aims at developing the treatment strategy based on a patient's genetic profile. This contrasts with the traditional “one-size-fits-all” approach, which assigns the same treatments to patients with the same diseases. Personalized approaches have been one of the most sought-after endeavors in the field due to their numerous advantages such as improving outcomes and reducing side effects,^4^ especially in oncology, where several biomarkers could lead to drastically different treatment plans.^3^ Despite the promise to understand the relations among treatments, diseases, high-dimensional genomics profiles, and the various outcomes, large-scale experiments in combinatorial complexity are required to investigate these relationships.^123^ ML provides valuable tools to facilitate this process.

#### Drug response prediction

It is known that the same small-molecule drug could have various response levels given different genomic profiles. For example, an anticancer drug has a different response to different tumors. Thus, it is crucial to generate an accurate response profile given drug-genomics profile pairs. However, to experimentally test each combination of available drugs and cell-line genomics profiles is prohibitively expensive.

An ML model can be used to predict a drug's response in a diverse set of cell lines *in silico*. An accurate ML model can greatly narrow down the drug screening space and reduce experimental costs and resources. Various models have been proposed to improve the accuracy, such as matrix factorization,^124^ VAEs,^125^ ensemble learning,^126^ similarity network model,^127^ and feature selection.^128^ While promising, one challenge is that the current public database has a limited number of drugs and genomics profiles tested, focusing on a small set of tissues or approved drug classes. It is often difficult for a model to generalize automatically to new contexts such as novel cell types and structurally diverse drugs with limited samples. For realistic adoption, ML models that can generalize to new domains given only a few labeled data points are thus highly desirable. This problem fits well with the few-shot meta-learning regime. Recently, Ma et al.^129^ tackled the few-shot drug response prediction problem. They apply model-agnostic meta-learning to learn from screening data of a set of tissues to generalize to new contexts such as new tissue types and pre-clinical studies in mice.^130^ In addition to accurate prediction, for a domain scientist to adopt the usage, it is also important to allow understanding of how the ML model makes the drug response prediction and what drug response mechanism is leveraged by it. Motivated by this, Kuenzi et al.^131^ firstly apply visible neural networks^121^ in the drug response prediction context by generating potential mechanisms and validating them through experiments using CRISPR, *in vitro* screening, and patient-derived tissue cultures.

##### Machine learning formulation

**Figure 7:**
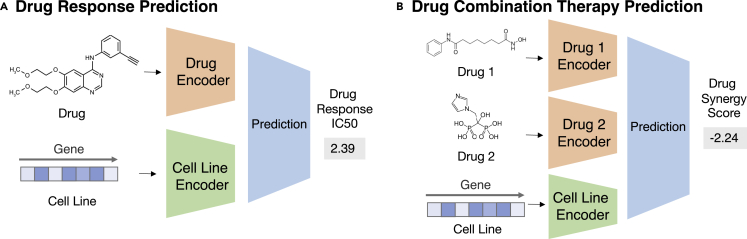
Task illustrations for the theme “improving context-specific drug response” (A) A drug encoder and a cell-line encoder produce embeddings for drug and cell line, respectively, which are then fed into a predictor to estimate drug response (see “drug response prediction”). (B) Drug encoders first map two drugs into embedding, and a cell-line encoder maps a cell line into embeddings. Three embeddings are then fed into a predictor for drug synergy scores (see “drug combination therapy prediction”).

Given a pair of drug compound molecular structures and gene expression profiles of the cell line, predict the drug response in this context. The task is illustrated in Figure 7A.

#### Drug combination therapy prediction

Drug combination therapy, also called cocktails, can expand the use of existing drugs, improve outcomes, and reduce side effects. For example, drug cocktails can modulate multiple targets to provide a novel mechanism of action in cancer treatments. Also, by reducing dosages for each drug, it may be possible to reduce adverse effects. However, screening the entire space of possible drug combinations and various cell lines is not feasible experimentally.

ML that can predict synergistic responses given the drug pair and the genomic profile for a cell line can prove valuable. Classical ML methods such as naive Bayes^132^ and random forests^133^ have shown initial success on independent external data. Deep learning methods such as DNNs^134^ and deep belief networks^135^ have shown improved performance. Integration with multi-omics data on cell lines has also further improved the performance, such as microRNA expression and proteomic features.^136^ Similar to drug response prediction, one important challenge is to transfer across tissue types and drug classes. Kim et al.^137^ pioneer this direction by conducting transfer learning to adapt models trained on data-rich tissues such as brain and breast tissues to understudied tissues such as bone and prostate tissues.

##### Machine learning formulation

Given a combination of drug compound structures and a cell line's genomics profile, predict the combination response. The task is illustrated in Figure 7B.

### Improving efficacy and delivery of gene therapy

Gene therapy is an emerging therapeutics class, which delivers nucleic acid instruction into patients’ cells to prevent or cure disease. These instructions include (1) replacing disease-causing genes with healthy ones, (2) turning off genes that cause diseases, and (3) inserting genes to produce disease-fighting proteins. Special vehicles called vectors are used to deliver these instructions (cargoes) into the cells and induce sufficient therapeutic effects. Many choices exist, such as naked DNA, virus, and nanoparticles. Virus vectors have become popular due to their natural ability to directly enter cells and replicate their genetic material. Despite the promise, numerous challenges still exist in reaching the expected effect, such as the host immune response, viral vector toxicity, and off-target effects. In recent years, ML tools have been shown to help tackle many of these challenges.

#### CRISPR on-target outcome prediction

CRISPR/Cas9 is a biotechnology that can edit genes in a precise location. It allows the correction of genetic defects to treat disease and provides a tool with which to alter the genome and to study gene function. CRISPR/Cas9 is a system with two important players. Cas9 protein is an enzyme that can cut through DNA, where the CRISPR sequence guides the cut location. The guide RNA sequence (gRNA) determines the specificity for the target DNA sequence in the CRISPR sequence. While existing CRISPR mostly make edits by small deletions, it is also under active research to carry out repair which, after cutting, a DNA template is provided to fill in the missing part of the gene. In theory, CRISPR can correctly edit the target DNA sequence and even restore a normal copy, but in reality the outcome varies significantly given different gRNAs.^138^ It has been shown that the outcome is decided by factors such as gRNA secondary structure and chromatin accessibility.^139^ Some of the desirable outcomes include insertion/deletion length, indel diversity, and the fraction of insertions/frameshifts. Thus, it is crucial to design a gRNA sequence such that the CRISPR/Cas system can achieve its effect on the designated target (also called on-target).

ML methods that can accurately predict the on-target outcome given the gRNA would facilitate the gRNA design process. Many classic ML methods have been investigated to predict various repair outcomes given gRNA sequence, such as linear models,^140^^,^^141^ support vector machines,^142^ and random forests.^143^ However, they do not capture the high-order non-linearity of gRNA features. Deep learning models that apply CNNs to automatically learn gRNA features show further improved performance.^144^^,^^145^ Despite the promise, numerous challenges still exist. For example, ML models are data-hungry. There is only a limited set of data with CRISPR knockout experiments, affecting the model's generalizability to new contexts such as new tissues. Besides, current models can only predict outcome while being incapable of generating the mechanism of how this gRNA sequence leads to the CRISPR outcome. For this high-stake biotechnology explainability is crucial, as an unexplained adverse effect could be detrimental for ML-designed gRNA sequence.

##### Machine learning formulation

**Figure 8:**
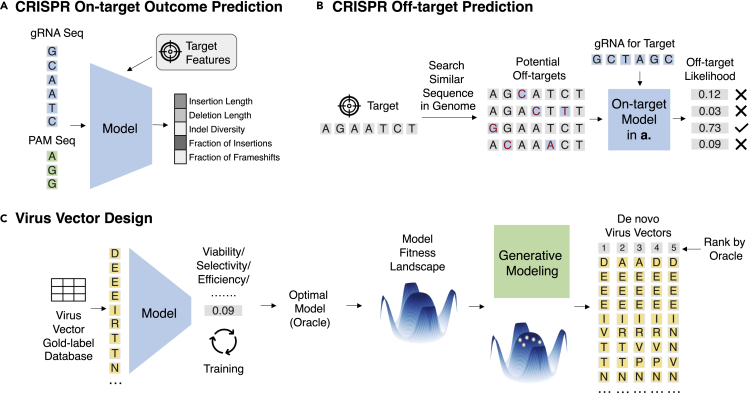
Task illustrations for the theme “improving efficacy and delivery of gene therapy” (A) A model predicts various gene-editing outcomes given the gRNA sequence and the target DNA features (see “CRISPR on-target outcome prediction”). (B) First, a model search through similar sequences to the target DNA sequence in the candidate genome and generate a list of potential off-target DNA sequences. Next, an on-target model predicts whether the gRNA sequence can affect these potential DNA sequences. The ones that have high on-target effects are considered potential off-targets (see “CRISPR off-target prediction”). (C) An optimal model (oracle function) is first obtained by training on a gold-label database. Next, a generative model generates *de novo* virus vectors potent in the oracle fitness landscape (see “virus vector design”).

With a fixed target, given the gRNA sequence and other auxiliary information such as target gene expression and epigenetic profile, predict its on-target repair outcome. The task is illustrated in Figure 8A.

#### CRISPR off-target prediction

As CRISPR can cut any region that matches the gRNA, it can potentially cut through similar off-target regions, leading to significant adverse effects. This is a major hurdle for CRISPR techniques for clinical implementations.^146^ Similar to on-target prediction, the off-target prediction is to predict whether gRNA could cause off-target effects. In contrast to on-target, where we have a fixed given DNA region, off-target prediction requires identifying potential off-target regions from the entire genome. Thus, the first step is to search and narrow down a set of potential hits using alignment algorithms and distance measures.^147^^,^^148^ Next, given the targets and the gRNA, a model needs to score the putative target-gRNA pair. The model also needs to aggregate these scores, since one gRNA usually has multiple putative off-targets. Various heuristics aggregation methods have been proposed and implemented.^149^, ^150^, ^151^, ^152^

Listgarten et al.^149^, ^150^, ^151^, ^152^ introduce ML for off-target prediction and show evidence of improved performance. They adopt a two-layer boosted regression tree where the first layer scores each gRNA-target pairs and the second layer aggregates the scores. Building upon this work, Lin and Wong^153^ apply CNN on a fused DNA-gRNA pair representation and achieve improved performance. There are still many open questions. For example, current ML approaches consist of two-stage approaches with first a heuristic search and then scoring. An end-to-end model that can automatically generate *de novo* candidate off-target sequences could be beneficial. Also, similar to the on-target prediction, as data of richer contexts such as different cell, tissue, and organism types become available, more sophisticated models that can generalize well on all contexts would be ideal.

##### Machine learning formulation

Given the gRNA sequence and the off-target DNA sequence, predict its off-target effect. The task is illustrated in Figure 8B.

#### Virus vector design

To deliver gene therapy instructions to cells and induce therapeutic effects, virus vectors are used as vehicles. The design of the virus vector is thus crucial. The recent development of adeno-associated virus (AAV) capsid vectors has led to a surge in gene therapy due to its favorable tropism, immunogenicity, and manufacturability properties.^154^ However, there are still unsolved challenges, mainly regarding the undesirable properties of natural AAV forms. For example, up to 50%–70% of humans are immune to the natural AAV vector, which means the human immune system would destroy it without delivering it to the targeted cells.^155^ This means that those patients are not able to receive gene therapies. Thus, designing functional variants of AAV capsids that can escape the immune system is crucial. Similarly, it would be ideal to design AAV variants with higher efficiency and selectivity to the tissue target of interest.

The standard method to generate new AAV variants is through “directed evolution” with limited diversity, most still similar to natural AAV. However, this is very time- and resource-intensive while the resulting yields are also low (<1%). Recently, Bryant et al.^156^ became the first to develop an ML-based framework to generate AAV variants that can escape the immune system with a >50% yield rate. They first train an ensemble neural network that aggregates DNN, CNN, and RNN using customized data collection to assign accurate viability scores given an AAV from diverse sources. They then sample iteratively on the predictor viability landscape to obtain a set of highly viable AAVs. Many opportunities remain open for machine-aided AAV design.^157^ For example, this framework can be easily extended to other targets in addition to the immune system viability, such as tissue selectivity, if a high-capacity ML property predictor can be constructed. Further improvements could be made by alternative generative strategies such as reinforcement learning or VAEs.

##### Machine learning formulation

Given a set of virus sequences and their labels for a property X, obtain an accurate predictor oracle and conduct various generation modeling to generate *de novo* virus variants with a high score in X and high diversity. The task is illustrated in Figure 8C.

## Machine learning for genomics in clinical studies

After a therapeutic is shown to have efficacy in the wet lab, it is further evaluated in animals and then on humans in full-scale clinical trials. ML can facilitate this process using genomics data. We review the following three themes. In this section we first study the long-standing problem of translating results from animals to humans and show that ML can enable better translation by better characterization of the molecular differences. We then review ML techniques to curate a better patient cohort to which the therapeutic can be applied, as it can greatly affect the clinical trial outcome. Last, we survey alternative ML techniques called causal inference to augment clinical trials in cases where traditional trials are not ethical or are difficult to conduct.

### Translating pre-clinical animal models to humans

Before therapeutics move into trials on humans, they are validated through extensive animal model experiments (pre-clinical studies). However, despite successful pre-clinical studies, more than 85% of early trials for novel drugs fail to translate to humans.^158^ One of the main factors for this failure is the gap between animal and human biology and physiology. Animal models do not mimic the human disease condition. However, by comparing large-scale omics data between animals and humans, we can identify translatable features and use ML to align animal and human models.

#### Animal-to-human translation

One of the central questions of animal-to-human translation is the following. If a study establishes relations between phenotypes and genotypes based on interventions in animals, do these relations persist in humans? Conventional computational methods construct cross-species pairs (CSPs) and compare the pair's molecular profile to find differential expression.^159^ Despite identifying several differential features associated with the disease, these methods often do not accurately translate to humans. One of the reasons is that human diseases are present simultaneously due to modulation on multiple pathways while the mouse model is an idealistic isolated system with the sole influence from the target disease. This gap of comorbidities may bias and occlude the true differential features. ML could help because it is good at modeling non-linear systems,^160^ although explainability methods are sought after to make sense of the non-linear relations.

To formulate it in ML, the genotype-phenotype relations can be captured by a computational model that builds upon an animal's molecular profile (such as using gene expression data to predict disease phenotypes). We can then evaluate the trained computational model to human molecular profiles (test set) and find out whether the model can accurately predict human phenotypes. A large ML challenge called SBV-IMPROVER was conducted to predict protein phosphorylation on human cells from rat cells using genomics and transcriptomics data under 52 stimulation conditions.^161^ A wide range of ML approaches such as DNNs, trees, and support vector machines have been applied and have shown promising extrapolation performance to humans.

However, the aforementioned works directly adopt ML models trained on mice and tested on humans, while we know human data present characteristics different from those of mouse data. This poses a challenge for ML, since the ML model often suffers from the out-of-distribution generalizability issue. Thus, it is crucial to explicitly model this out-of-distribution property by identifying and leveraging translatable features between animals and humans. Notably, Brubaker et al.^162^ propose a semi-supervised technique that integrates unsupervised modeling of human disease-context datasets into the supervised component that trains on mouse data. In addition, works that directly train on CSPs have been proposed. For example, Normand et al.^163^ aim to identify translatable genes. For every gene, they compute the disease effect size for humans and rats in each CSP and apply linear models to fit them. After fitting, they use the mean of the linear model as the predicted human effect size for this gene. They show improved gene selection by up to 50%. Another important avenue of research is computational network models, which leverage existing biological knowledge about system-level signaling pathways and mechanistic models. They have been shown to identify transferrable biomarkers and predictable pathways.^164^^,^^165^ The animal-to-human translation problem is tackling a central problem in ML: domain adaptation. Basically, it requires the model to bridge the gap between the source domain and target domain, where sources are data that are labeled while the target domain only has input but no label.^166^ Opportunities to leverage advanced domain adaptation techniques to this problem remain open. Another challenge is that data availability is a hurdle to applying ML for this problem, since it requires new data for every animal model and disease indication.

##### Machine learning formulation

**Figure 9:**
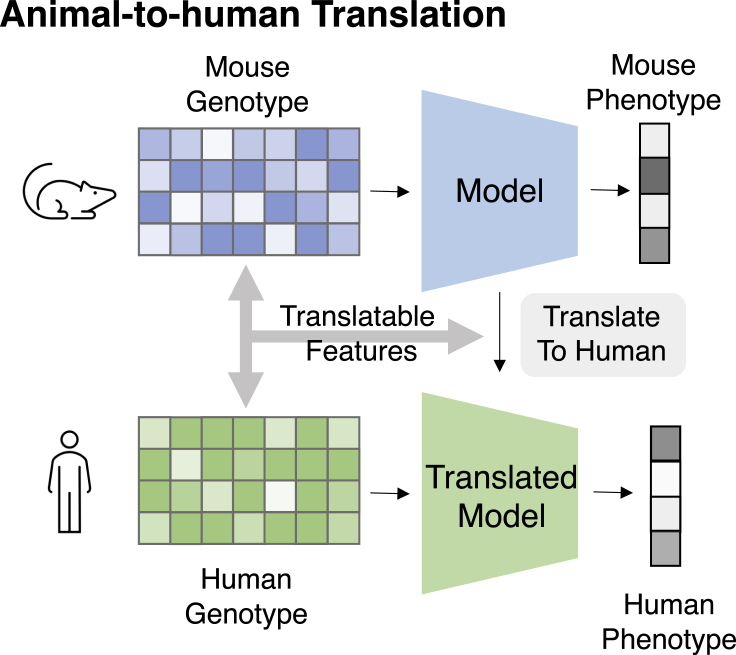
Task illustration for the theme “translating pre-clinical animal models to humans” A model first obtains translatable features between mouse and human by comparing their genotypes. Next, a predictor model is trained to predict phenotype given the mouse genotype. Given the translatable features, the predictor is augmented and makes predictions on human genotypes (see “animal-to-human translation”).

Given genotype-phenotype data of animals and only the genotype data of humans, train the model to fit the phenotype from the genotype and transfer this model to humans. The task is illustrated in Figure 9.

### Curating high-quality cohorts

To study the efficacy of therapeutics in the intended or target patient groups, a clinical trial requires a precise and accurate patient population in each arm.^167^ However, due to the heterogeneity of patients, it may be difficult to recruit and enroll appropriate patients. ML can help characterize important factors for the primary endpoints and quickly identify them in patients by predicting patient molecular profiles.

#### Patient stratification/disease subtyping

Patient stratification in clinical trials is designed to create more homogeneous subgroups with respect to risk of outcome or other important variables that might impact the validity of the comparison between treatment arms. Some therapeutics may be highly effective in one patient subgroup and have a weak or even no effect in other subgroups. In the absence of appropriate stratification in heterogeneous patient populations, the average treatment effect across all patients will obscure potentially strong effects in a subpopulation. Conventional stratification methods rely on manual rules on a few available features such as clinical genomics biomarkers, but this might ignore signals arising from rich patient data. ML can potentially identify these important criteria for stratification based on heterogeneous data sources such as genomics profiles, patient demographics, and medical history.

This problem can be approached as unsupervised learning, whereby we strive to obtain representations that can easily group each sample of gene expression into distinct categories and claim each category as a subtype. These methods include clustering,^168^^,^^169^ gene network stratification,^170^ and matrix factorization.^171^ Integrating existing biomedical knowledge can be useful. Notably, Chen et al.^172^ propose a DNN-based clustering method in which a supervised constraint on gold-standard subtype knowledge is included. As the data are high-dimensional and heterogeneous, fusing diverse data sources can help models to obtain a comprehensive picture of the patient conditions and lead to more accurate and granular stratification. Notably, Wang et al.^173^ aggregate mRNA expression, DNA methylation, and microRNA data through similarity network fusion for cancer subtyping. Similarly, Jurmeister et al.^174^ leverage DNA methylation profiles to subtype lung cancers using DNN and Li et al.^175^ apply topological data analysis on the patient-patient similarity network constructed from each patient's genotype and EHR data to identify type 2 diabetes subgroups. Despite the accuracy, these methods suffer from interpretability, which is especially important in patient stratification. A black-box stratification output based on a complex model is often not trustworthy for practitioners to adopt. Explainable ML models are thus highly desirable. Decision-tree methods are a classical interpretable ML model. For example, Valdes et al.^176^ apply a boosted decision-tree method with high accuracy compared with a standard decision tree while still providing clues for how the model makes the accurate prediction/stratification. For detailed discussion on the challenge for explainability, we refer readers to a later section (“discussion: open challenges and opportunities”).

##### Machine learning formulation

**Figure 10:**
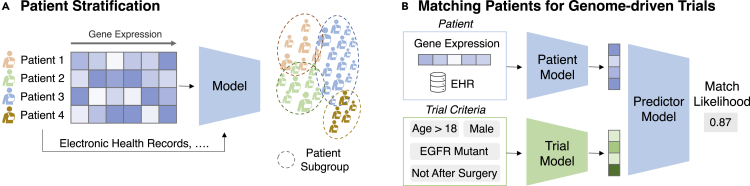
Task illustrations for the theme “curating high-quality cohort” (A) Given the patient's gene expressions and EHRs, a model clusters them into subgroups (see “patient stratification/disease subtyping”). (B) A patient model obtains patient embedding from his/her gene expression and EHR. A trial model obtains trial embedding based on trial criteria. A predictor predicts whether this patient is fit for enrollment in the given trial (see “matching patients for genome-driven trials”).

Given the gene expression and other auxiliary information for a set of patients, produce criteria for patient stratification. The task is illustrated in Figure 10A.

#### Matching patients for genome-driven trials

Clinical trials suffer from difficulties in recruiting a sufficient number of patients. Mendelsohn et al.^177^ report that 40% of trials fail to complete accrual in the National Clinical Trial Network and Murthy et al.^178^ show that less than 2% of adults with cancer enroll in any clinical trials. Many factors can prevent successful enrollment, such as limited awareness of available trials and ineffective methods to identify eligible patients in the traditional manual matching system.^179^

Automated patient-trial matching could be desirable to increase enrollment by taking account into the heterogeneous patient data and trial eligibility criteria. Conventional patient-trial matching methods rely on rule-based annotations. For example, Tao et al.^180^ conducted a real-world outcome analysis using an automatic patient-trial matching alert system based on the patient's genomic biomarkers and showed improved results compared with manual matching. However, these are based on heuristics matching rules, which often omit useful information in rich patient data. These complex data modalities call for DNNs. Notably, Bustod and Pertusa^181^ introduce DNN to generate eligibility criteria, but no matching is done. Recently, advanced ML methods have been proposed to leverage the EHR data from patients to match the eligibility criteria of a trial. Zhang et al.^182^ pioneered the study on using advanced pre-trained Bidirectional Encoder Representations from Transformers model for encoding trial protocols into sentence embedding, and used a hierarchical embedding model to represent patient longitudinal EHR. Building upon this work, Gao et al.^183^ propose a multi-granularity memory network to encode structured patient medical codes and use a convolutional highway network to encode trial eligibility criteria, showing significant improvement over previous conventional rule-based methods. However, genomics information is not included. Methods that fuse genome and EHR data to represent patients could further improve matching efficiency in genome-driven trials.

##### Machine learning formulation

Given a pair of patient data (e.g., genomics, EHR) and trial eligibility criteria (text description), predict the matching likelihood. The task is illustrated in Figure 10B.

### Inferring causal effects

Clinical trials study treatment efficacy on humans. Numerous unmeasured confounders can lead to a biased conclusion about the efficacy. To eliminate these confounders, randomization is conducted such that the control and treatment groups would have an equal distribution of confounders. This way, the comparative effect is not due to unmeasured confounders. However, this requires that the control group receives an alternative therapy (e.g., placebo or standard of care). In many studies, it is difficult or unethical to devise and assign placebos/treatments. In these cases, observational studies can be used to study the correlations between exposure (e.g., smoking) and an outcome (e.g., cancer). However, these studies are typically subjected to unmeasured confounding, since no randomization is introduced. Recent methods in causal inference provide alternative ways to conduct randomization through genomics information.

#### Mendelian randomization

Mendelian randomization (MR) uses genes as a mediator for robust causal inference.^184^ The key is that genetic information is not modified by post-natal events and is thus not susceptible to confounders. If a gene is associated with the exposure and the outcome via the exposure (i.e., vertical pleiotropy), we can use genes as an instrumental variable to simulate randomization. For example, we know that CHRNA5 genes are associated with smoking levels. Thus, we can use the CHRNA5 status to group patients and estimate the comparative effect on outcome (e.g., mortality). This process has a tremendous impact as it can bypass clinical trials, add support for trials, and serve as validation for drug targets.^185^^,^^186^ Regression analysis is usually conducted to calculate the effects. Despite the promise, challenges remain for more advanced ML and causal inference methods. One challenge is that in some cases, the assumption of vertical pleiotropy does not hold. For example, the genes can associate with the outcome through another pathway (i.e., horizontal pleiotropy).^187^ This requires customized probabilistic models and larger sample size for statistically significant estimation.^188^ The underlying causal pathways among exposures, genes, and outcomes are usually not obvious in many cases due to limited knowledge. A large-scale causal pathway could not only help protect MR from horizontal pleiotropy by knowing when it could be the case but also allows more accurate causal inference with advanced methods by the inclusion of other genes or selection of alternative genes as the instrument variable. The main challenge in obtaining this putative causal map is that different models can contradict conclusions given the same dataset. Hemani et al.^189^ apply a mixture-of-experts random forest framework to reduce the false discovery rate on a set of genome-wide association studies data to construct a large-scale causal map of human genome and phenotype and show its usefulness in MR.

##### Machine learning formulation

**Figure 11:**
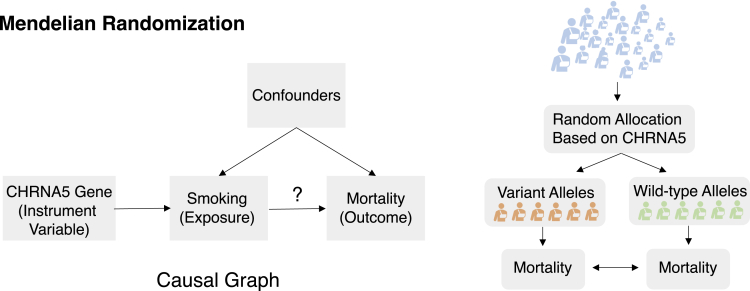
Task illustrations for the theme “inferring causal effects” Left panel: Mendelian randomization relies on using a gene biomarker (e.g., CHRNA5) as an instrumental variable to measure the effect of exposure to the outcome as it is not affected by confounders, and it serves as a proxy for exposure by directly comparing the effect of the gene on the outcome. Right panel: patients are first grouped based on the CHRNA5 gene. One group contains variant alleles and another contains wild-type alleles. The mortality rate can then be calculated within each group and compared with ascertained risks. If the risk is high, we conclude that the exposure causes the outcome (see “Mendelian randomization”).

Given observation data of the genomic factor, exposure, outcome, and other auxiliary information, formulate or identify the causal relations among them and compute the effect of the exposure to the outcome. The task is illustrated in Figure 11.

## Machine learning for genomics in post-market studies

After a therapeutic is evaluated in clinical trials and approved for marketing, numerous studies monitor its efficacy and safety when used in clinical practice. These studies contain important and often unknown information about therapeutics that was not evident before regulatory approval. This section reviews how ML can mine through a large corpus of texts and identify useful signals for post-market surveillance.

### Mining real-world evidence

After therapeutics are approved and used to treat patients, voluminous documentation is generated in the EHR system, insurance billing system, and scientific literature. These are called real-world data. The analyses of these data are called real-world evidence. They contain important insights about therapeutics, such as patients' drug responses given different patient characteristics. They can also shed light on disease mechanism of action, the novel phenotype for a target gene, and so forth. However, free-form texts are notoriously difficult to process. Natural language processing (NLP) technology can be helpful to mine insights from these texts. Next, we describe two specific tasks involving real-world evidence, namely, clinical notes and scientific literature.

#### Clinical text biomarker mining

An EHR has rich information about the patient and records a wide range of patient's vitals and disease courses after treatments. This information is critical for post-market research, from which an actionable hypothesis can be drawn. However, the structured EHR data do not cover the entire picture of a patient. The majority of important variables can only be found in the clinical notes,^190^ such as next-generation sequencing (NGS) status, PDL1 (immunotherapy) status, treatment change, and so forth. These variables can directly facilitate predictive model building to support clinical decision making or increase the power of disease-gene-drug associations to better understand the drug. However, conventional human annotations are costly and time consuming, and are not scalable.

Automatic processing of clinical notes of patients using ML can facilitate this process. For example, Guan et al.^191^ use bidirectional LSTMs to extract NGS-related information in a patient's genetics report and classify documents to the treatment-change and no-treatment-change groups. However, the clinical text is very messy and filled with typos and jargon (e.g., acronyms). Standard NLP techniques do not work. Also, clinical text often requires clinical annotations. Specialized ML models are required, such as transfer learning techniques that learn a sufficient clinical note representation through large-scale self-supervised learning on clinical notes and fine-tuning on a task of interest with a small number of annotations.^14^^,^^192^ Another challenge is that clinical notes are long, especially for intensive care unit patients. This length poses special constraint, even for the advanced efficient transformer models. Huang et al.^193^ introduced a hierarchical scheme to drastically improve efficiency. They applied the model to classify PDL1 and NGS status and used an attention mechanism to provide clues for which parts of a text provide these variables.

##### Machine learning formulation

**Figure 12:**
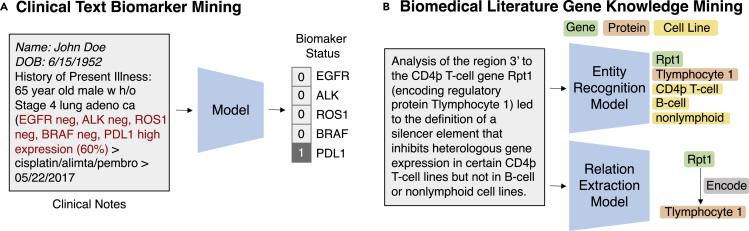
Task illustrations for the theme “mining real-world evidence” (A) A model predicts genomic biomarker status given a patient's clinical notes (see “clinical text biomarker mining”). (B) A model recognizes entities in the literature and extracts relations among these entities (see “biomedical literature gene knowledge mining”). The text in panel (A) is from Huang et al.;^193^ the text in panel (B) is from Zhu et al.^194^

Given a clinical note document, predict the genomic biomarker variable of interest. The task is illustrated in Figure 12A.

#### Mining of biomedical literature gene knowledge

One key question in post-market research is to find evidence about a therapeutic’s response to diseases given patient characteristics such as genomic biomarkers. This has several important applications such as validation of therapeutic efficacy, identification of potential off-label genes/diseases for drug repurposing, and detection of therapeutic candidates' adverse events when treating patients, using some genomic biomarkers. It also serves as important complementary information for target discovery. This summarized information about drug-gene and disease-gene relations is usually reported and published in the scientific literature. Manual annotations are infeasible due to the exponential number of new articles published every day.

Conventional methods are rule-based^210^ and dictionary-based.^211^ They both rely on hand-crafted rules/features to construct query biomedical text templates and search through the papers to find sentences that match these templates.^212^ However, these hand-crafted features require extensive domain knowledge and are difficult to keep up to date with new literature. The limited flexibility leads to the omission of potential newly discovered drug-gene/drug-disease pairs. Recent advances in name entity recognition and relation detection through deep learning can automatically learn from a large corpus to obtain an optimal set of features without human engineering and have shown strong performances.^213^ This can be formulated as a model to recognize drugs, genes, and disease terms, and to detect drug-gene or drug-disease relation types given a set of documents. Numerous ML methods have been developed for biomedical named entity recognition/relation extraction. For example, Limsopatham and Collier^214^ use bidirectional LSTM to predict the name entity label for each word with character-level embedding. Zhu et al.^194^ use an n-gram based CNN to capture local context around each word for improved prediction.

On relation extraction, in addition to the CNN^215^ and RNN^216^ architecture, Zhang et al.^217^ propose a hybrid model that integrates a CNN on a syntax dependency tree and an RNN on the sentence encodings for improved biomedical relation prediction. Zhang et al.^218^ apply a graph CNN on the syntax dependency tree of a sentence and show improved relation extraction. ML models require large amounts of label annotations as training data, which can be difficult to obtain. Distant supervision borrows information from a large-scale knowledge base to automatically create labels so that it does not require labeled corpora, which reduces manual annotation efforts. Lamurias et al.^219^ apply a distant-learning-based pipeline that predicts microRNA-gene relations. Recently, BioBERT extended BERT^14^ to pre-train on a large-scale biomedical scientific literature corpus and fine-tune it on numerous downstream tasks and has shown sound performance in benchmarking tasks such as biomedical named entity recognition and relation extraction.

##### Machine learning formulation

Given a document from literature, extract the drug-gene and drug-disease terms, and predict the interaction types from the text. The task is illustrated in Figure 12B.

## Discussion: Open challenges and opportunities

This survey provides an overview of research in the intersection of ML, genomics, and therapeutic developments. It is our view that ML has the potential to revolutionize the use of genomics in therapeutics development, as we have presented a diverse set of such applications in preceding sections. However, numerous challenges remain. Here, we discuss these challenges and the associated opportunities.

### Distribution shifts

ML models work well when the training and deployment data follow the same data distribution. However, in real-world use in genomics and therapeutics ML, many problems experience distribution shifts whereby the deployment environment and the data generated from it are different from the training stage. As ML models often tend to fit spurious correlations hidden in the training data, when these correlations are unseen in the testing data due to distribution shift, the model performance would decrease significantly.^220^ For example, training happens given the available batches of gene expression data in brain tissue. The resulting model is required to predict a new experiment with bone tissue. A robust model is required to pick up signals that are invariant between brain and bone tissues during training on the brain tissue. Another example is to train on animal model transcriptomics and predict the phenotype of human models. Similarly, the model should not learn to use signals that are only present in animals for prediction, as these signals are unseen in the human models. Thus, a model must generalize to out-of-distribution. Distribution shifts have been a long-standing challenge in ML, and a large body of work in model robustness and domain adaptation could be applied to genomics to improve generalizability.^221^ For instance, Brbić et al.^222^ utilize the meta-learning technique to generalize to novel single-cell experiments.

### Learning from small datasets

Biological data are generated through expensive experiments. This means that many tasks only have a minimal number of labeled data points. For example, there are usually only a few drug response data points for new therapeutics. However, standard ML models, especially deep learning models, are data-hungry. Thus, how to make an ML model learn given only a few examples is crucial. Transfer learning can learn from a large body of existing labeled data points and transfer it to the downstream task with limited data points.^14^ However, they usually still require a reasonable number of training data points. Given only a few data points, few-shot learning methods such as model-agnostic meta-learning (MAML)^130^ and prototypical networks^223^ learning from other related tasks using a few examples have shown strong promise. Recently, Ma et al.^129^ have successfully applied MAML to improve few-shot drug response prediction.

### Representation capacity

The key to successful ML models depends on the effective representation of the genome and other related biomedical entities that match biological motivation. For example, the current dominant ML model for DNA sequence is through CNN models. However, most successful usage only applies to short DNA sequences generated from pre-defined pre-processing steps instead of a large fraction of the whole-genome sequence, which could allow a model to tap into crucial information of long-range gene regulatory dependencies.^53^^,^^55^ RNN and transformers are also only able to take in medium-length inputs, in contrast to more than $O\left(10^{6}\right)$ SNPs per genome. This also means that the number of input features can be orders of magnitude larger than the number of data points, a well-known ML challenge called the curse of dimensionality. Furthermore, the general ML models are often developed for image and text data without any biological motivations. Thus, to model the human genome and the complicated regulation among genes, a domain-motivated model that captures interactions among extremely long-range high-dimensional features is needed. Initial attempts for domain-motivated representation learning have been made. For instance, Romero et al.^224^ propose a parameter prediction network that reduces the number of free parameters for DNN to alleviate the aforementioned curse of dimensionality issue and shows improved patient stratification given $10^{6}$ SNPs. Ma et al.^121^ modify the neural network structure to simulate the hierarchical biological processes and explain pathways for phenotype prediction.

### Model trustworthiness

For an ML model to be used by domain scientists, the model prediction has to be trustworthy. This can happen on two levels. First, in addition to accurate prediction, the model prediction also needs to generate justification in terms of biomedical knowledge (“explanation”). However, current ML models focus on improving model prediction accuracy. Toward the goal of explanation, ML models need to encode biomedical knowledge. This can be potentially achieved by integrating biological knowledge graphs^225^ and applying the graph explainability method.^226^

The second level is on the quality of model prediction. Since ML models are not error free, it is important to alert the users or abstain from making predictions when the model is not confident. Uncertainty quantification or model abstention around the model prediction can alleviate this problem. Recently, Hie et al.^102^ used Gaussian processes to generate uncertainty scores of compound bioactivity, protein fluorescence, and single-cell transcriptomic imputation, which were shown to guide the experimental and validation loop. Integrating the explanation into human workflows and promoting human trust in AI also requires special attention, as recent works show that directly providing AI explanation to humans can confuse the human observer and degrade the performance.^227^

### Fairness

ML models can manifest bias in the training data. It has been shown that ML models do not work equally well on all subpopulations. These algorithmic biases could have significant social and ethical consequences. For example, Martin et al.^228^ find that 79% of genomic data are from patients of European descent, even though they constitute only 16% of the world's population. Due to differences in allele frequencies and effect sizes across populations, ML models that perform well on the discovery population generally have much lower accuracy and are worse predictors in other populations. As most discovery to date is performed with European-ancestry cohorts, predictive models may exacerbate health disparities, since they will not be available for or have lower utility in African and Hispanic ancestry populations. Similarly, most studies focus on common diseases, whereas experimental data on rare diseases are often limited. These imbalances against minorities require specialized ML techniques. The fairness in ML is defined to make the prediction independent of protected variables such as race, gender, and sexual orientation.^229^ Recent works have been proposed to ensure this criterion in the clinical ML domain.^230^ However, fairness research of ML for the genomic domain is still lacking.

### Data linking and integration

An individual has a diverse set of data modalities, such as genomics, transcriptomics, proteomics, EHRs, and social-economic data. Current ML approaches focus on developing methods for a single data modality, whereas to fully capture the comprehensive data types around individuals could potentially unlock new biological insights and actionable hypotheses. One of the reasons for the limited integration is the lack of data access that connects these heterogeneous data types. As large-scale efforts such as UK Biobank,^231^ which connects in-depth genetic and EHR information about a patient, become available, new ML methods designed to consider this heterogeneity are needed. Indeed, recent studies have discovered novel insights by applying ML to linked genomics and EHR data.^232^, ^233^, ^234^ Another challenge in data linking is that the availability of data also varies across data modalities. For example, plasma samples are abundant, whereas there are only a few cerebrospinal fluid samples. This leads to a high percentage of missing data for a large cohort of patients. Thus, how to handle missing data is a common challenge in this setting. Classic techniques include heuristic imputations based on similar samples.^175^ However, they often rely heavily on the assumption that similar samples must have similar values for all features. Recently, ML methods that explicitly model the variety of missing data through masking^235^ or non-linear imputation^236^ when building architectures have also shown initial promise.

### Genomics data privacy

Abundant genomics data and annotations are generated every day. Aggregation of these data and annotations can tremendously benefit ML models. However, these are usually considered private assets for individuals and contain sensitive private information, and thus are not shareable directly. Techniques to anonymize and de-identify these data using differential privacy can potentially enable genomics data sharing.^237^ In addition, recent advances in federated learning techniques allow ML model training on aggregated data without sharing data.^238^

## Conclusion

We have conducted a comprehensive review of the literature on ML applications for genomics in therapeutics development. We systematically identify diverse ML applications in genomics and provide pointers to the latest methods and resources. For ML researchers, we show that most of these applications have problems that remain unsolved, thus providing many technical challenges for ML method innovations. We also provide concise ML problem formulation to help ML researchers to approach these tasks. For biomedical researchers, we pinpoint a large set of diverse use cases of ML applications, which they can extend to novel use cases. We also introduce the popular ML models and their corresponding use cases in genomics data.

In conclusion, this survey provides an in-depth research summary of the intersection of ML, genomics, and therapeutic developments. We hope that this survey can lead to a deeper understanding of this interdisciplinary domain between ML and genomics and broaden the collaboration across these two communities. As a common belief that the future of medicine is personalized, understanding the therapeutic tasks with ML methods on genomics data is the key that will lead to ultimate breakthroughs in drug discovery and development. We hope that this survey will help to bridge the gap between genomics and ML domains.

## References

[1] Hieter P., Boguski M. (1997). Functional genomics: it’s all how you read it. Science.

[2] Wong M.-L., Licinio J. (2004). From monoamines to genomic targets: a paradigm shift for drug discovery in depression. Nat. Rev. Drug Discov..

[3] Chin L., Andersen J.N., Futreal P.A. (2011). Cancer genomics: from discovery science to personalized medicine. Nat. Med..

[4] Hamburg M.A., Collins F.S. (2010). The path to personalized medicine. New Engl. J. Med..

[5] Makarova K.S., Haft D.H., Barrangou R., Brouns S.J., Charpentier E., Horvath P., Moineau S., Mojica F.J.M., Wolf Y.I., Yakunin A.F. (2011). Evolution and classification of the CRISPR–cas systems. Nat. Rev. Microbiol..

[6] Reuter J.A., Spacek D.V., Snyder M.P. (2015). High-throughput sequencing technologies. Mol. Cell.

[7] Heath A.P., Ferretti V., Agrawal S., An M., Angelakos J.C., Arya R., Bajari R., Baqar B., Barnowski J.H.B., Burt J. (2021). The NCI genomic data commons. Nat. Genet..

[8] Rinn J.L., Chang H.Y. (2012). Genome regulation by long noncoding RNAs. Annu. Rev. Biochem..

[9] Singal R., Ginder G.D. (1999). DNA methylation. J. Am. Soc. Hematol..

[10] Rogers J., Wall R. (1980). A mechanism for RNA splicing. Proc. Natl. Acad. Sci. U S A.

[11] Fu Y., Foden J.A., Khayter C., Maeder M.L., Reyon D., Joung J.K., Sander J.D. (2013). High-frequency off-target mutagenesis induced by CRISPR-Cas nucleases in human cells. Nat. Biotechnol..

[12] Corrigan-Curay J., Sacks L., Woodcock J. (2018). Real-world evidence and real-world data for evaluating drug safety and effectiveness. J. Am. Med. Assoc..

[13] Krizhevsky A., Sutskever I., Hinton G.E. (2012). ImageNet classification with deep convolutional neural networks. NeurIPS.

[14] Devlin J., Chang M.-W., Lee K., Toutanova K. (2019). Proceedings of NAACL-HLT 2019.

[15] Silver D., Huang A., Maddison C.J., Guez A., Sifre L., Van Den Driessche G., Schrittwieser J., Antonoglou I., Panneershelvam V., Lanctot M. (2016). Mastering the game of Go with deep neural networks and tree search. Nature.

[16] Stokes J.M., Yang K., Swanson K., Jin W., Cubillos-Ruiz A., M Donghia N., MacNair C.R., French S., Carfrae L.A., Bloom-Ackermann Z. (2020). A deep learning approach to antibiotic discovery. Cell.

[17] Senior A.W., Evans R., Jumper J., Kirkpatrick J., Sifre L., Green T., Qin C., Žídek A., Nelson A.W.R., Bridgland A. (2020). Improved protein structure prediction using potentials from deep learning. Nature.

[18] Leung M.K.K., Delong A., Alipanahi B., Frey B.J. (2015). Machine learning in genomic medicine: a review of computational problems and data sets. Proc. IEEE.

[19] Eraslan G., Avsec Ž., Gagneur J., Theis F.J. (2019). Deep learning: new computational modelling techniques for genomics. Nat. Rev. Genet..

[20] Zou J., Huss M., Abid A., Mohammadi P., Torkamani A., Telenti A. (2019). A primer on deep learning in genomics. Nat. Genet..

[21] Gaudelet T., Day B., Jamasb A.R., Soman J., Regep C., Liu G., Hayter J.B.R., Vickers R., Roberts C., Tang J. (2020). Utilising graph machine learning within drug discovery and development. arXiv.

[22] Piotrowska Z. (2017). A study of EGF816 and gefitinib in TKI-naïve EGFR-mutant non-small cell lung cancer. https://clinicaltrials.gov/ct2/show/NCT03292133.

[23] Hu W., Liu B., Gomes J., Zitnik M., Liang P., Pande V., Leskovec J. (2019). ICLR 2020.

[24] Tran H.T.N., Kok S.A., Chevrier M., Zhang X., Lee N.Y.S., Goh M., Chen J. (2020). A benchmark of batch-effect correction methods for single-cell RNA sequencing data. Genome Biol..

[25] Van Buuren S. (2018).

[26] Argelaguet R., Anna S.E.C., Oliver S., Marioni J.C. (2021). Computational principles and challenges in single-cell data integration. Nat. Biotechnol..

[27] Huang K., Fu T., Gao W., Zhao Y., Roohani Y., Leskovec J., Coley C.W., Xiao C., Sun J., Zitnik M. (2021). Therapeutics data commons: machine learning datasets and tasks for therapeutics. arXiv.

[28] Zitnik M., Nguyen F., Wang B., Leskovec J., Goldenberg A., Hoffman M.M. (2019). Machine learning for integrating data in biology and medicine: principles, practice, and opportunities. Inf. Fusion.

[29] Mitchell T.M. (1997).

[30] Rosenblatt F. (1961).

[31] LeCun Y., Bengio Y., Arbib M.A. (1995). The Handbook of Brain Theory and Neural Networks.

[32] De Mulder W., Bethard S., Moens M.-F. (2015). A survey on the application of recurrent neural networks to statistical language modeling. Comput. Speech Lang..

[33] Hochreiter S., Schmidhuber J. (1997). Long short-term memory. Neural Comput..

[34] Cho K., Van Merriënboer B., Bahdanau D., Bengio Y. (2014). Proceedings of SSST-8, Eighth Workshop on Syntax, Semantics and Structure in Statistical Translation.

[35] Vaswani A., Shazeer N., Parmar N., Uszkoreit J., Jones L., Gomez A.N., Kaiser L., Polosukhin I. (2017). 31st Conference on Neural Information Processing Systems (NIPS 2017).

[36] Rives A., Meier J., Sercu T., Goyal S., Lin Z., Liu J., Guo D., Ott M., Lawrence Zitnick C., Ma J., Fergus R. (2021). Biological structure and function emerge from scaling unsupervised learning to 250 million protein sequences. Proc. Natl. Acad. Sci. U S A.

[37] Huang K., Xiao C., Glass L.M., Sun J. (2020). MolTrans: molecular interaction transformer for drug target interaction prediction. Bioinformatics.

[38] Kitaev N., Kaiser Ł., Levskaya A. (2020). ICLR Proceedings.

[39] Kipf T.N., Welling M. (2017). ICLR Proceedings of 5th International Conference on Learning Representations.

[40] Huang K., Xiao C., Glass L.M., Zitnik M., Sun J. (2020). SkipGNN: predicting molecular interactions with skip-graph networks. Sci. Rep..

[41] Schütt K.T., Sauceda H.E., Kindermans P.-J., Tkatchenko A., Müller K.-R. (2018). SchNet—a deep learning architecture for molecules and materials. J. Chem. Phys..

[42] Kramer M.A. (1991). Nonlinear principal component analysis using autoassociative neural networks. AIChE J..

[43] Vincent P., Larochelle H., Lajoie I., Bengio Y., Manzagol P.-A., Bottou L. (2010). Stacked denoising autoencoders: learning useful representations in a deep network with a local denoising criterion. J. Machine Learn. Res..

[44] Kingma D.P., Welling M. (2013). Auto-encoding variational bayes. arXiv.

[45] Wittrock M.C. (1974). Learning as a generative process. Educ. Psychol..

[46] Goodfellow I.J., Pouget-Abadie J., Mirza M., Xu B., Warde-Farley D., Ozair S., Courville A., Bengio Y. (2020). Generative adversarial networks. Commun. ACM.

[47] Fu T., Xiao C., Sun J. (2020). AAAI.

[48] Jin W., Barzilay R., Jaakkola T. (2018). ICML.

[49] Jha A., Gazzara M.R., Barash Y. (2017). Integrative deep models for alternative splicing. Bioinformatics.

[50] Xiong H.Y., Alipanahi B., Lee L.J., Bretschneider H., Merico D., Yuen R.K.C., Hua Y., Gueroussov S., Najafabadi H.S., Hughes T.R. (2015). The human splicing code reveals new insights into the genetic determinants of disease. Science.

[51] Angermueller C., Lee H.J., Reik W., Stegle O. (2017). DeepCpG: accurate prediction of single-cell DNA methylation states using deep learning. Genome Biol..

[52] Liu F., Li H., Ren C., Bo X., Shu W. (2016). PEDLA: predicting enhancers with a deep learning-based algorithmic framework. Sci. Rep..

[53] Zhou J., Troyanskaya O.G. (2015). Predicting effects of noncoding variants with deep learning-based sequence model. Nat. Methods.

[54] Angermueller C., Pärnamaa T., Parts L., Stegle O. (2016). Deep learning for computational biology. Mol. Syst. Biol..

[55] Alipanahi B., Delong A., Weirauch M.T., Frey B.J. (2015). Predicting the sequence specificities of DNA-and RNA-binding proteins by deep learning. Nat. Biotechnol..

[56] Kircher M., Witten D.M., Jain P., O’Roak B.J., Cooper G.M., Shendure J. (2014). A general framework for estimating the relative pathogenicity of human genetic variants. Nat. Genet..

[57] Zeng H., Edwards M.D., Liu G., Gifford D.K. (2016). Convolutional neural network architectures for predicting DNA–protein binding. Bioinformatics.

[58] Kelley D.R., Snoek J., Rinn J.L. (2016). Basset: learning the regulatory code of the accessible genome with deep convolutional neural networks. Genome Res..

[59] Zhang Q., Zhu L., Huang D.-S. (2018). High-order convolutional neural network architecture for predicting DNA-protein binding sites. IEEE/ACM Trans. Comput. Biol. Bioinformatics.

[60] Cao Z., Zhang S. (2019). Simple tricks of convolutional neural network architectures improve DNA-protein binding prediction. Bioinformatics.

[61] Avsec Ž., Weilert M., Shrikumar A., Krueger S., Alexandari A., Dalal K., Fropf R., McAnany C., Gagneur J., Kundaje A. (2021). Base-resolution models of transcription-factor binding reveal soft motif syntax. Nat. Genet..

[62] He B., Bergenstråhle L., Stenbeck L., Abid A., Andersson A., Borg Å., Maaskola J., Lundeberg J., Zou J. (2020). Integrating spatial gene expression and breast tumour morphology via deep learning. Nat. Biomed. Eng..

[63] Robertson K.D. (2005). DNA methylation and human disease. Nat. Rev. Genet..

[64] Zhang W., Spector T.D., Deloukas P., Bell J.T., Engelhardt B.E. (2015). Predicting genome-wide DNA methylation using methylation marks, genomic position, and DNA regulatory elements. Genome Biol..

[65] Whitaker J.W., Chen Z., Wang W. (2015). Predicting the human epigenome from DNA motifs. Nat. Methods.

[66] Kapourani C.-A., Sanguinetti G. (2019). Melissa: Bayesian clustering and imputation of single-cell methylomes. Genome Biol..

[67] Levy J.J., Titus A.J., Petersen C.L., Chen Y., Salas L.A., Christensen B.C. (2020). MethylNet: an automated and modular deep learning approach for DNA methylation analysis. BMC Bioinformatics.

[68] Zou Q., Xing P., Wei L., Liu B. (2019). Gene2vec: gene subsequence embedding for prediction of mammalian n6-methyladenosine sites from mRNA. RNA.

[69] López-Bigas N., Audit B., Ouzounis C., Parra G., Guigó R. (2005). Are splicing mutations the most frequent cause of hereditary disease?. FEBS Lett..

[70] Gelfman S., Wang Q., McSweeney K.M., Ren Z., La Carpia F., Halvorsen M., Schoch K., Ratzon F., Heinzen E.L., Boland M.J. (2017). Annotating pathogenic non-coding variants in genic regions. Nat. Commun..

[71] Paggi J.M., Bejerano G. (2018). A sequence-based, deep learning model accurately predicts RNA splicing branchpoints. RNA.

[72] Jagadeesh K.A., Paggi J.M., James S.Y., Stenson P.D., Cooper D.N., Bernstein J.A., Bejerano G. (2019). S-cap extends pathogenicity prediction to genetic variants that affect RNA splicing. Nat. Genet..

[73] Ståhl P.L., Salmén F., Vickovic S., Lundmark A., Fernández Navarro J., Magnusson J., Giacomello S., Asp M., Westholm J.O., Huss M. (2016). Visualization and analysis of gene expression in tissue sections by spatial transcriptomics. Science.

[74] Levy-Jurgenson A., Tekpli X., Kristensen V.N., Yakhini Z. (2020). Spatial transcriptomics inferred from pathology whole-slide images links tumor heterogeneity to survival in breast and lung cancer. Sci. Rep..

[75] Bergenstråhle L., He B., Bergenstråhle J., Andersson A., Lundeberg J., Zou J., Maaskola J. (2020). Super-resolved spatial transcriptomics by deep data fusion. bioRxiv.

[76] Egeblad M., Nakasone E.S., Werb Z. (2010). Tumors as organs: complex tissues that interface with the entire organism. Dev. Cell.

[77] Avila Cobos F., Vandesompele J., Mestdagh P., De Preter K. (2018). Computational deconvolution of transcriptomics data from mixed cell populations. Bioinformatics.

[78] Newman A.M., Liu C.L., Green M.R., Gentles A.J., Feng W., Xu Y., Hoang C.D., Diehn M., Alizadeh A.A. (2015). Robust enumeration of cell subsets from tissue expression profiles. Nat. Methods.

[79] Menden K., Marouf M., Oller S., Dalmia A., Magruder D.S., Kloiber K., Heutink P., Bonn S. (2020). Deep learning-based cell composition analysis from tissue expression profiles. Sci. Adv..

[80] Andersson A., Bergenstråhle J., Asp M., Bergenstråhle L., Jurek A., Fernández Navarro J., Lundeberg J. (2020). Single-cell and spatial transcriptomics enables probabilistic inference of cell type topography. Commun. Biol..

[81] Su J., Song Q. (2021). DSTG: deconvoluting spatial transcriptomics data through graph-based artificial intelligence. Brief. Bioinform..

[82] Arazo E., Ortego D., Albert P., O.’Connor N., McGuinness K. (2019). Proceedings of the 36th International Conference on Machine Learning.

[83] Haury A.-C., Mordelet F., Vera-Licona P., Vert J.-P. (2012). TIGRESS: Trustful Inference of Gene REgulation using Stability Selection. BMC Syst. Biol..

[84] Huynh-Thu V.A., Irrthum A., Wehenkel L., Geurts P. (2010). Inferring regulatory networks from expression data using tree-based methods. PLoS One.

[85] Moerman T., Santos S.A., González-Blas C.B., Simm J., Moreau Y., Aerts J., Aerts S. (2019). GRNBoost2 and Arboreto: efficient and scalable inference of gene regulatory networks. Bioinformatics.

[86] Shrivastava H., Zhang X., Aluru S., Song L. (2020). GRNUlar: gene regulatory network reconstruction using unrolled algorithm from single cell RNA-sequencing data. bioRxiv.

[87] DePristo M.A., Banks E., Poplin R., Garimella K.V., Maguire J.R., Hartl C., Philippakis A.A., Angel G.D., Rivas M.A. (2011). A framework for variation discovery and genotyping using next-generation DNA sequencing data. Nat. Genet..

[88] Poplin R., Chang P.-C., Alexander D., Schwartz S., Colthurst T., Ku A., Newburger D., Dijamco J., Nguyen N., Afshar P.T. (2018). A universal SNP and small-indel variant caller using deep neural networks. Nat. Biotechnol..

[89] Luo R., Sedlazeck F.J., Lam T.-W., Schatz M.C. (2019). A multi-task convolutional deep neural network for variant calling in single molecule sequencing. Nat. Commun..

[90] Luo R., Wong C.-L., Wong Y.-S., Tang C.-I., Liu C.-M., Leung C.-M., Lam T.-W. (2020). Exploring the limit of using a deep neural network on pileup data for germline variant calling. Nat. Machine Intelligence.

[91] Zook J.M., McDaniel J., Olson N.D., Wagner J., Parikh H., Heaton H., Irvine S.A., Trigg L., Truty R., Cory Y. (2019). An open resource for accurately benchmarking small variant and reference calls. Nat. Biotechnol..

[92] Delaneau O., Zagury J.-F., Marchini J. (2013). Improved whole-chromosome phasing for disease and population genetic studies. Nat. Methods.

[93] Zhao Y., Wang K., Wang W.-L., Yin T.-T., Dong W.-Q., Xu C.-J. (2019). A high-throughput SNP discovery strategy for RNA-seq data. BMC Genomics.

[94] Wolfien M., Klatt D., Salybekov A.A., Masaaki, Komatsu-Horii M., Gaebel R., Philippou-Massier J., Schrinner E., Akimaru H., Akimaru E. (2020). Hematopoietic stem-cell senescence and myocardial repair-coronary artery disease genotype/phenotype analysis of post-mi myocardial regeneration response induced by CABG/CD133+ bone marrow hematopoietic stem cell treatment in rct perfect phase 3. EBioMedicine.

[95] Quang D., Chen Y., Xie X. (2015). DANN: a deep learning approach for annotating the pathogenicity of genetic variants. Bioinformatics.

[96] Kooperberg C., LeBlanc M., Obenchain V. (2010). Risk prediction using genome-wide association studies. Genet. Epidemiol..

[97] Paré G., Mao S., Deng W.Q. (2017). A machine-learning heuristic to improve gene score prediction of polygenic traits. Sci.Rep..

[98] Isgut M., Sun J., Quyyumi A.A., Gibson G. (2021). Highly elevated polygenic risk scores are better predictors of myocardial infarction risk early in life than later. Genome Med..

[99] Zhou J., Theesfeld C.L., Yao K., Chen K.M., Wong A.K., Troyanskaya O.G. (2018). Deep learning sequence-based ab initio prediction of variant effects on expression and disease risk. Nat. Genet..

[100] Zhou J., Park C.Y., Theesfeld C.L., Wong A.K., Yuan Y., Scheckel C., Fak J.J., Funk J., Yao K. (2019). Whole-genome deep-learning analysis identifies contribution of noncoding mutations to autism risk. Nat. Genet..

[101] Li X., Wang Y., Ruiz R. (2020). A survey on sparse learning models for feature selection. IEEE Trans. Cybern..

[102] Hie B., Bryson B.D., Berger B. (2020). Leveraging uncertainty in machine learning accelerates biological discovery and design. Cell Syst..

[103] Vickers P.J. (2013). Challenges and opportunities in the treatment of rare diseases. Drug Discov. World.

[104] Yin B., Balvert M., van der Spek R.A.A., Dutilh B.E., Bohté S., Veldink J., Schönhuth A. (2019). Using the structure of genome data in the design of deep neural networks for predicting amyotrophic lateral sclerosis from genotype. Bioinformatics.

[105] Chawla N.V., Bowyer K.W., Hall L.O., Kegelmeyer W.P. (2002). SMOTE: synthetic minority over-sampling technique. J. Artif. intelligence Res..

[106] He H., Bai Y., Garcia E.A., Li S. (2008). 2008 IEEE International Joint Conference on Neural Networks (IEEE World Congress on Computational Intelligence).

[107] Bej S., Davtyan N., Wolfien M., Nassar M., Wolkenhauer O. (2021). LoRAS: an oversampling approach for imbalanced datasets. Machine Learn..

[108] Cui L., Biswal S., Glass L.M., Lever G., Sun J., Xiao C. (2020).

[109] Taroni J.N., Grayson P.C., Hu Q., Eddy S., Kretzler M., Merkel P.A., Greene C.S. (2019). MultiPLIER: a transfer learning framework for transcriptomics reveals systemic features of rare disease. Cell Syst..

[110] Wolfe C.J., Kohane I.S., Butte A.J. (2005). Systematic survey reveals general applicability of “guilt-by-association” within gene coexpression networks. BMC Bioinformatics.

[111] Köhler S., Bauer S., Horn D., Robinson P.N. (2008). Walking the interactome for prioritization of candidate disease genes. Am. J. Hum. Genet..

[112] Tranchevent L.-C., Ardeshirdavani A., ElShal S., Alcaide D., Aerts J., Auboeuf D., Moreau Y. (2016). Candidate gene prioritization with endeavour. Nucleic Acids Res..

[113] Luo P., Li Y., Tian L.-P., Wu F.-X. (2019). Enhancing the prediction of disease–gene associations with multimodal deep learning. Bioinformatics.

[114] Cáceres J.J., Paccanaro A. (2019). Disease gene prediction for molecularly uncharacterized diseases. PLoS Comput. Biol..

[115] Park Y.-J., Tuzhilin A. (2008). RecSys ’08.

[116] Subramanian A., Tamayo P., Mootha V.K., Mukherjee S., Ebert B.L., Gillette M.A., Paulovich A., Pomeroy S.L., Golub T.R. (2005). Gene set enrichment analysis: a knowledge-based approach for interpreting genome-wide expression profiles. Proc. Natl. Acad. Sci. U S A.

[117] Tarca A.L., Draghici S., Khatri P., Hassan S.S., Mittal P., Kim J.S., Kim C.J., Kusanovic J.P., Romero R. (2009). A novel signaling pathway impact analysis. Bioinformatics.

[118] Ozerov I.V., Lezhnina K.V., Izumchenko E., Artemov A.V., Medintsev S., Vanhaelen Q., Aliper A., Vijg J., Osipov A.N., Labat I. (2016). In silico pathway activation network decomposition analysis (iPANDA) as a method for biomarker development. Nat. Commun..

[119] Reyna M.A., Haan D., Paczkowska M., Verbeke L.P.C., Vazquez M., Kahraman A., Pulido-Tamayo S., Barenboim J., Wadi L., Dhingra P. (2020). Pathway and network analysis of more than 2500 whole cancer genomes. Nat. Commun..

[120] Karr J.R., Sanghvi J.C., Macklin D.N., Gutschow M.V., Jacobs J.M., Bolival B., Assad-Garcia N., Glass J.I., Covert M.W. (2012). A whole-cell computational model predicts phenotype from genotype. Cell.

[121] Ma J., Ku Yu M., Fong S., Ono K., Sage E., Demchak B., Sharan R., Ideker T. (2018). Using deep learning to model the hierarchical structure and function of a cell. Nat. Methods.

[122] Elmarakeby H.A., Hwang J., Liu D., AlDubayan S.H., Salari K., Richter C., Arnoff T.E., Park J., Hahn W.C., Van Allen E. (2020). Biologically informed deep neural network for prostate cancer classification and discovery. bioRxiv.

[123] Menden M.P., Wang D., Mason M.J., Szalai B., Bulusu K.C., Guan Y., Yu T., Kang J., Jeon M., Wolfinger R. (2019). Community assessment to advance computational prediction of cancer drug combinations in a pharmacogenomic screen. Nat. Commun..

[124] Ammad-Ud-Din M., Khan S.A., Malani D., Murumägi A., Kallioniemi O., Aittokallio T., Kaski S. (2016). Drug response prediction by inferring pathway-response associations with kernelized bayesian matrix factorization. Bioinformatics.

[125] Rampášek L., Hidru D., Smirnov P., Haibe-Kains B., Goldenberg A. (2019). Dr.VAE: improving drug response prediction via modeling of drug perturbation effects. Bioinformatics.

[126] Tan M., Fırat Özgül O., Bardak B., Ekşioğlu I., Sabuncuoğlu S. (2019). Drug response prediction by ensemble learning and drug-induced gene expression signatures. Genomics.

[127] Zhang N., Wang H., Fang Y., Wang J., Zheng X., Liu X.S. (2015). Predicting anticancer drug responses using a dual-layer integrated cell line-drug network model. PLoS Comput. Biol..

[128] Ali M., Aittokallio T. (2019). Machine learning and feature selection for drug response prediction in precision oncology applications. Biophysical Rev..

[129] Ma J., Fong S.H., Luo Y., Bakkenist C.J., Shen J.P., Mourragui S., Wessels L.F.A., Hafner M., Sharan R., Peng J. (2021). Few-shot learning creates predictive models of drug response that translate from high-throughput screens to individual patients. Nat. Cancer.

[130] Finn C., Abbeel P., Levine S., Precup D., Teh Y.W. (2017).

[131] Kuenzi B.M., Park J., Fong S.H., Sanchez K.S., Lee J., Kreisberg J.F., Ma J., Ideker T. (2020). Predicting drug response and synergy using a deep learning model of human cancer cells. Cancer Cell.

[132] Li P., Huang C., Fu Y., Wang J., Wu Z., Ru J., Zheng C., Guo Z., Chen X., Zhou W. (2015). Large-scale exploration and analysis of drug combinations. Bioinformatics.

[133] Wildenhain J., Spitzer M., Dolma S., Jarvik N., White R., Roy M., Griffiths E., Bellows D.S., Wright G.D., Tyers M. (2015). Prediction of synergism from chemical-genetic interactions by machine learning. Cell Syst..

[134] Preuer K., Lewis R.P.I., Hochreiter S., Bender A., Bulusu K.C., Klambauer G. (2018). DeepSynergy: predicting anti-cancer drug synergy with deep learning. Bioinformatics.

[135] Chen G., Tsoi A., Xu H., Zheng W.J. (2018). Predict effective drug combination by deep belief network and ontology fingerprints. J. Biomed. Inform..

[136] Xia F., Shukla M., Brettin T., Garcia-Cardona C., Cohn J., Allen J.E., Maslov S., Holbeck S.L., Doroshow J.H., Evrard Y.A. (2018). Predicting tumor cell line response to drug pairs with deep learning. BMC Bioinformatics.

[137] Kim Y., Zheng S., Tang J., Zheng W.J., Li Z., Jiang X. (2021). Anticancer drug synergy prediction in understudied tissues using transfer learning. J. Am. Med. Inform. Assoc..

[138] Cong L., Ran F.A., Cox D., Lin S., Barretto R., Habib N., Hsu P.D., Wu X., Jiang W. (2013). Multiplex genome engineering using CRISPR/cas systems. Science.

[139] Jensen K.T., Fløe L., Petersen T.S., Huang J., Xu F., Bolund L., Luo Y., Lin L. (2017). Chromatin accessibility and guide sequence secondary structure affect CRISPR-Cas9 gene editing efficiency. FEBS Lett..

[140] Labuhn M., Adams F.F., Ng M., Knoess S., Schambach A., Charpentier E.M., Schwarzer A., Mateo J.L., Klusmann J.-H., Heckl D. (2018). Refined sgRNA efficacy prediction improves large-and small-scale CRISPR-Cas9 applications. Nucleic Acids Res..

[141] Moreno-Mateos M.A., Vejnar C.E., Beaudoin J.-D., Fernandez J.P., Mis E.K., Khokha M.K., Giraldez A.J. (2015). CrisprScan: designing highly efficient sgRNAs for CRISPR-Cas9 targeting in vivo. Nat. Methods.

[142] Chari R., Mali P., Moosburner M., Church G.M. (2015). Unraveling CRISPR-Cas9 genome engineering parameters via a library-on-library approach. Nat. Methods.

[143] Wilson L.O.W., Reti D., O’Brien A.R., Dunne R.A., Bauer D.C. (2018). High activity target-site identification using phenotypic independent CRISPR-Cas9 core functionality. CRISPR J..

[144] Chuai G., Ma H., Yan J., Chen M., Hong N., Xue D., Zhou C., Zhu C., Chen K., Duan B. (2018). DeepCRISPR: optimized CRISPR guide RNA design by deep learning. Genome Biol..

[145] Kim H.K., Min S., Song M., Jung S., Choi J.W., Kim Y., Lee S., Yoon S., Kim H.H. (2018). Deep learning improves prediction of CRISPR-CPF1 guide RNA activity. Nat. Biotechnol..

[146] Zhang X.-H., Tee L.Y., Wang X.-G., Huang Q.-S., Yang S.-H. (2015). Off-target effects in CRISPR/Cas9-mediated genome engineering. Mol. Therapy-Nucleic Acids.

[147] Heigwer F., Kerr G., Boutros M. (2014). E-crisp: fast CRISPR target site identification. Nat. Methods.

[148] Bae S., Park J., Kim J.-S. (2014). Cas-OFFinder: a fast and versatile algorithm that searches for potential off-target sites of Cas9 RNA-guided endonucleases. Bioinformatics.

[149] Hsu P.D., Scott D.A., Weinstein J.A., Ran F.A., Konermann S., Agarwala V., Li Y., Fine E.J., Wu X. (2013). DNA targeting specificity of RNA-guided Cas9 nucleases. Nat. Biotechnol..

[150] Haeussler M., Schönig K., Eckert H., Eschstruth A., Mianné J., Renaud J.-B., Schneider-Maunoury S., Shkumatava A., Teboul L., Kent J. (2016). Evaluation of off-target and on-target scoring algorithms and integration into the guide RNA selection tool CRISPOR. Genome Biol..

[151] Cradick T.J., Qiu P., Lee C.M., Fine E.J., Bao G. (2014). COSMID: a web-based tool for identifying and validating CRISPR/Cas off-target sites. Mol. Therapy-Nucleic Acids.

[152] Listgarten J., Weinstein M., Kleinstiver B.P., Sousa A.A., Joung J.K., Crawford J., Gao K., Hoang L., Elibol M., Doench J.G. (2018). Prediction of off-target activities for the end-to-end design of CRISPR guide RNAs. Nat. Biomed. Eng..

[153] Lin J., Wong K.-C. (2018). Off-target predictions in CRISPR-Cas9 gene editing using deep learning. Bioinformatics.

[154] Daya S., Berns K.I. (2008). Gene therapy using adeno-associated virus vectors. Clin. Microbiol. Rev..

[155] Chirmule N., Propert K.J., Magosin S.A., Qian Y., Qian R., Wilson J.M. (1999). Immune responses to adenovirus and adeno-associated virus in humans. Gene Ther..

[156] Bryant D.H., Bashir A., Sinai S., Jain N.K., Ogden P.J., Riley P.F., Church G.M., Colwell L.J., Kelsic E.D. (2021). Deep diversification of an AAV capsid protein by machine learning. Nat. Biotechnol..

[157] Kelsic E.D., Church G.M. (2019). Challenges and opportunities of machine-guided capsid engineering for gene therapy. Cell Gene Ther. Insights.

[158] Mak I.W.Y., Evaniew N., Ghert M. (2014). Lost in translation: animal models and clinical trials in cancer treatment. Am. J. Translational Res..

[159] Naqvi S., Godfrey A.K., Hughes J.F., Goodheart M.L., Mitchell R.N., Page D.C. (2019). Conservation, acquisition, and functional impact of sex-biased gene expression in mammals. Science.

[160] Hassaine A., Salimi-Khorshidi G., Canoy D., Rahimi K. (2020). Untangling the complexity of multimorbidity with machine learning. Mech. ageing Dev..

[161] Rhrissorrakrai K., Belcastro V., Bilal E., Norel R., Poussin C., Mathis C., Dulize R.H.J., Ivanov N.V., Alexopoulos L., Rice J.J. (2015). Understanding the limits of animal models as predictors of human biology: lessons learned from the SBV improver species translation challenge. Bioinformatics.

[162] Brubaker D.K., Proctor E.A., Haigis K.M., Lauffenburger D.A. (2019). Computational translation of genomic responses from experimental model systems to humans. PLoS Comput. Biol..

[163] Normand R., Du W., Briller M., Gaujoux R., Starosvetsky E., Ziv-Kenet A., Shalev-Malul G., Tibshirani R.J., Shen-Orr S.S. (2018). Found in translation: a machine learning model for mouse-to-human inference. Nat. Methods.

[164] Yao V., Kaletsky R., Keyes W., Mor D.E., Wong A.K., Sohrabi S., Murphy C.T., Troyanskaya O.G. (2018). An integrative tissue-network approach to identify and test human disease genes. Nat. Biotechnol..

[165] Blais E.M., Rawls K.D., Dougherty B.V., Li Z.I., Kolling G.L., Ye P., Wallqvist A., Papin J.A. (2017). Reconciled rat and human metabolic networks for comparative toxicogenomics and biomarker predictions. Nat. Commun..

[166] Wang M., Deng W. (2018). Deep visual domain adaptation: a survey. Neurocomputing.

[167] Trusheim M.R., Berndt E.R., Douglas F.L. (2007). Stratified medicine: strategic and economic implications of combining drugs and clinical biomarkers. Nat. Rev. Drug Discov..

[168] Shen R., Wang S., Mo Q. (2013). Sparse integrative clustering of multiple omics data sets. Ann. Appl. Stat..

[169] Witten D.M., Tibshirani R. (2010). A framework for feature selection in clustering. J. Am. Stat. Assoc..

[170] Hofree M., Shen J.P., Carter H., Gross A., Ideker T. (2013). Network-based stratification of tumor mutations. Nat. Methods.

[171] Gao Y., Church G. (2005). Improving molecular cancer class discovery through sparse non-negative matrix factorization. Bioinformatics.

[172] Chen R., Yang L., Goodison S., Sun Y. (2020). Deep-learning approach to identifying cancer subtypes using high-dimensional genomic data. Bioinformatics.

[173] Wang B., Mezlini A.M., Demir F., Fiume M., Tu Z., Brudno M., Haibe-Kains B., Goldenberg A. (2014). Similarity network fusion for aggregating data types on a genomic scale. Nat. Methods.

[174] Jurmeister P., Bockmayr M., Seegerer P., Bockmayr T., Treue D., Montavon G., Vollbrecht C., Arnold A., Teichmann D., Bressem K. (2019). Machine learning analysis of DNA methylation profiles distinguishes primary lung squamous cell carcinomas from head and neck metastases. Sci. Transl. Med..

[175] Li L., Cheng W.-Y., Glicksberg B.S., Gottesman O., Tamler R., Chen R., Bottinger E.P., Dudley J.T. (2015). Identification of type 2 diabetes subgroups through topological analysis of patient similarity. Sci. Transl. Med..

[176] Valdes G., Luna J.M., Eaton E., Simone C.B., Ungar L.H., Solberg T.D. (2016). MediBoost: a patient stratification tool for interpretable decision making in the era of precision medicine. Sci. Rep..

[177] Mendelsohn J., Moses H.L., Nass S.J. (2010). A National Cancer Clinical Trials System for the 21st Century: Reinvigorating the NCI Cooperative Group Program.

[178] Murthy V.H., Krumholz H.M., Gross C.P. (2004). Participation in cancer clinical trials: race-, sex-, and age-based disparities. JAMA.

[179] Lee S.J.C., Murphy C.C., Geiger A.M., Gerber D.E., Cox J.V., Nair R., Skinner C.S. (2019). Conceptual model for accrual to cancer clinical trials. J. Clin. Oncol..

[180] Tao J.J., Eubank M.H., Schram A.M., Cangemi N., Pamer E., Rosen E.Y., Schultz N., Chakravarty D., Philip J. (2019). Real-world outcomes of an automated physician support system for genome-driven oncology. JCO Precision Oncol..

[181] Bustos A., Pertusa A. (2018). Learning eligibility in cancer clinical trials using deep neural networks. Appl. Sci..

[182] Zhang X., Xiao C., Glass L.M., Sun J. (2020). WWW ’20: Proceedings of the Web Conference 2020.

[183] Gao J., Xiao C., Glass L.M., Sun J. (2020). Proceedings of the 26th ACM SIGKDD International Conference on Knowledge Discovery & Data Mining.

[184] Smith G.D., Ebrahim S. (2003). ‘Mendelian randomization’: can genetic epidemiology contribute to understanding environmental determinants of disease?. Int. J. Epidemiol..

[185] Emdin C.A., Khera A.V., Kathiresan S. (2017). Mendelian randomization. JAMA.

[186] Ference B.A., Yoo W., Alesh I., Mahajan N., Mirowska K.K., Mewada A., Kahn J., Afonso L., Williams K.A., Flack J.M. (2012). Effect of long-term exposure to lower low-density lipoprotein cholesterol beginning early in life on the risk of coronary heart disease: a Mendelian randomization analysis. J. Am. Coll. Cardiol..

[187] Verbanck M., Chen C.-Y., Neale B., Do R. (2018). Detection of widespread horizontal pleiotropy in causal relationships inferred from Mendelian randomization between complex traits and diseases. Nat. Genet..

[188] Cho Y., Haycock P.C., Sanderson E., Gaunt T.R., Zheng J., Morris A.P., Smith G.D., Hemani G. (2020). Exploiting horizontal pleiotropy to search for causal pathways within a Mendelian randomization framework. Nat. Commun..

[189] Hemani G., Bowden J., Haycock P., Zheng J., Davis O., Flach P., Gaunt T., Smith G.D. (2017). Automating Mendelian randomization through machine learning to construct a putative causal map of the human phenome. bioRxiv.

[190] Boag W., Doss D., Naumann T., Szolovits P. (2018). What’s in a note? Unpacking predictive value in clinical note representations. AMIA Summits Transl. Sci. Proc..

[191] Guan M., Cho S., Petro R., Zhang W., Pasche B., Topaloglu U. (2019). Natural language processing and recurrent network models for identifying genomic mutation-associated cancer treatment change from patient progress notes. JAMIA Open.

[192] Huang K., Altosaar J., Ranganath R. (2021). CHIL 2020 Workshop.

[193] Huang K., Garapati S., Rich A.S. (2020). NeurIPS ML4H Workshop.

[194] Zhu Q., Li X., Conesa A., Pereira C. (2018). Gram-CNN: a deep learning approach with local context for named entity recognition in biomedical text. Bioinformatics.

[195] Levy J.J., Titus A.J., Salas L.A., Christensen B.C. (2019). PyMethylProcess—convenient high-throughput preprocessing workflow for DNA methylation data. Bioinformatics.

[196] Harrow J., Frankish A., Gonzalez J.M., Tapanari E., Diekhans M., Kokocinski F., Aken B.L., Barrell D., Zadissa A., Searle S. (2012). GenCode: the reference human genome annotation for the encode project. Genome Res..

[197] Weinstein J.N., Collisson E.A., Mills G.B., Shaw K.R.M., Ozenberger B.A., Ellrott K., Shmulevich I., Sander C., Stuart J.M. (2013). The cancer genome atlas pan-cancer analysis project. Nat. Genet..

[198] Avila Cobos F., Alquicira-Hernandez J., Powell J.E., Mestdagh P., De Preter K. (2020). Benchmarking of cell type deconvolution pipelines for transcriptomics data. Nat. Commun..

[199] Chen J., Li X., Zhong H., Meng Y., Du H. (2019). Systematic comparison of germline variant calling pipelines cross multiple next-generation sequencers. Sci. Rep..

[200] Landrum M.J., Lee J.M., Riley G.R., Jang W., Rubinstein W.S., Church D.M., Maglott D.R. (2014). ClinVar: public archive of relationships among sequence variation and human phenotype. Nucleic Acids Res..

[201] Piñero J., Bravo À., Queralt-Rosinach N., Gutiérrez-Sacristán A., Deu-Pons J., Centeno E., García-García J., Sanz F., Furlong L.I. (2016). DisGeNET: a comprehensive platform integrating information on human disease-associated genes and variants. Nucleic Acids Res..

[202] Fabregat A., Jupe S., Matthews L., Sidiropoulos K., Gillespie M., Garapati P., Haw R., Jassal B., Korninger F., May B. (2018). The reactome pathway knowledgebase. Nucleic Acids Res..

[203] Yang W., Soares J., Greninger P., Edelman E.J., Lightfoot H., Forbes S., Bindal N., Beare D., Smith J.A., Thompson I.R. (2012). Genomics of drug sensitivity in cancer (GDSC): a resource for therapeutic biomarker discovery in cancer cells. Nucleic Acids Res..

[204] Liu H., Zhang W., Zou B., Wang J., Deng Y., Deng L. (2020). DrugCombDB: a comprehensive database of drug combinations toward the discovery of combinatorial therapy. Nucleic Acids Res..

[205] Leenay R.T., Aghazadeh A., Hiatt J., Tse D., Roth T.L., Apathy R., Shifrut E., Hultquist J.F., Krogan N., Wu Z. (2019). Large dataset enables prediction of repair after CRISPR-Cas9 editing in primary t cells. Nat. Biotechnol..

[206] Störtz F., Minary P. (2021). CRISPRSQL: a novel database platform for CRISPR/Cas off-target cleavage assays. Nucleic Acids Res..

[207] Poussin C., Mathis C., Alexopoulos L.G., Messinis D.E., Dulize R.H.J., Belcastro V., Melas I.N., Sakellaropoulos T., Rhrissorrakrai K., Bilal E. (2014). The species translation challenge—a systems biology perspective on human and rat bronchial epithelial cells. Sci. Data.

[208] Curtis C., Shah S.P., Chin S.F., Turashvili G., Rueda O.M., Dunning M.J., Speed D., Lynch A.G., Samarajiwa S., Yuan Y. (2012). . The genomic and transcriptomic architecture of 2,000 breast tumours reveals novel subgroups. Nature.

[209] Pyysalo S., Ginter F., Heimonen J., Björne J., Boberg J., Järvinen J., Salakoski T. (2007). BioInfer: a corpus for information extraction in the biomedical domain. BMC Bioinformatics.

[210] Tsai R.T.-H.N., Sung C.-L., Dai H.-J., Hung H.-C., Sung T.-Y., Hsu W.-L. (2006). NERBio: using selected word conjunctions, term normalization, and global patterns to improve biomedical named entity recognition. BMC Bioinformatics.

[211] Hirschman L., Morgan A.A., Yeh A.S. (2002). Rutabaga by any other name: extracting biological names. J. Biomed. Inform..

[212] Davis A.P., Wiegers T.C., Roberts P.M., King B.L., Lay J.M., Lennon-Hopkins K., Sciaky D., Johnson R., Keating H., Greene N. (2013). A CTD-Pfizer collaboration: manual curation of 88 000 scientific articles text mined for drug-disease and drug-phenotype interactions. Database.

[213] Nasar Z., Jaffry S.W., Malik M.K. (2018). Information extraction from scientific articles: a survey. Scientometrics.

[214] Limsopatham N., Collier N. (2016). Proceedings of the Fifth Workshop on Building and Evaluating Resources for Biomedical Text Mining (BioTxtM2016).

[215] Zhao Z., Yang Z., Luo L., Lin H., Wang J. (2016). Drug drug interaction extraction from biomedical literature using syntax convolutional neural network. Bioinformatics.

[216] Zhang Y., Zheng W., Lin H., Wang J., Yang Z., Dumontier M. (2018). Drug-drug interaction extraction via hierarchical RNNS on sequence and shortest dependency paths. Bioinformatics.

[217] Zhang Y., Lin H., Yang Z., Wang J., Zhang S., Sun Y., Yang L. (2018). A hybrid model based on neural networks for biomedical relation extraction. J. Biomed. Inform..

[218] Zhang Y., Qi P., Manning C.D. (2018). Proceedings of the 2018 Conference on Empirical Methods in Natural Language Processing.

[219] Lamurias A., Clarke L.A., Couto F.M. (2017). Extracting microRNA-gene relations from biomedical literature using distant supervision. PLoS One.

[220] Arjovsky M., Bottou L., Gulrajani I., Lopez-Paz D. (2020). Invariant risk minimization. arXiv.

[221] Moreno-Torres J.G., Raeder T., Alaiz-Rodríguez R., Chawla N.V., Herrera F. (2012). A unifying view on dataset shift in classification. Pattern Recogn..

[222] Brbić M., Zitnik M., Wang S., Pisco A.O., Altman R.B., Darmanis S., Leskovec J. (2020). MARS: discovering novel cell types across heterogeneous single-cell experiments. Nat. Methods.

[223] Snell J., Swersky K., Zemel R.S. (2017). 31st Conference on Neural Information Processing Systems (NIPS 2017).

[224] Romero A., Luc Carrier P., Erraqabi A., Sylvain T., Auvolat A., Dejoie E., Legault Marc-A., Dubé M.-P., Hussin J.G., Bengio Y. (2017). International Conference on Representation Learning (ICLR), 2017.

[225] Himmelstein D.S., Lizee A., Hessler C., Brueggeman L., Chen S.L., Hadley D., Green A., Khankhanian P., Baranzini S.E. (2017). Systematic integration of biomedical knowledge prioritizes drugs for repurposing. eLife.

[226] Ying R., Bourgeois D., You J., Zitnik M., Leskovec J. (2019). GNNexplainer: generating explanations for graph neural networks. NeurIPS.

[227] Bansal G., Wu T., Zhu J., Fok R., Nushi B., Kamar E., Ribeiro M.T., Weld D.S. (2021). CHI ’21.

[228] Martin A.R., Kanai M., Kamatani Y., Okada Y., Neale B.M., Daly M.J. (2018). Hidden ‘risk’ in polygenic scores: clinical use today could exacerbate health disparities. bioRxiv.

[229] Barocas S., Hardt M., Narayanan A. (2017).

[230] Pierson E., Cutler D.M., Leskovec J., Mullainathan S., Obermeyer Z. (2021). An algorithmic approach to reducing unexplained pain disparities in underserved populations. Nat. Med..

[231] Canela-Xandri O., Rawlik K., Tenesa A. (2018). An atlas of genetic associations in UK biobank. Nat. Genet..

[232] Shen L., Thompson P.M. (2019). Brain imaging genomics: integrated analysis and machine learning. Proc. IEEE.

[233] Willetts M., Hollowell S., Aslett L., Holmes C., Doherty A. (2018). Statistical machine learning of sleep and physical activity phenotypes from sensor data in 96,220 UK biobank participants. Sci. Rep..

[234] Bellot P., de Los Campos G., Pérez-Enciso M. (2018). Can deep learning improve genomic prediction of complex human traits?. Genetics.

[235] Che Z., Purushotham S., Cho K., Sontag D., Liu Y. (2018). Recurrent neural networks for multivariate time series with missing values. Sci. Rep..

[236] You J., Ma X., Ding D.Y., Kochenderfer M., Leskovec J. (2020). 34th Conference on Neural Information Processing Systems (NeurIPS 2020).

[237] Azencott C.-A. (2018). Machine learning and genomics: precision medicine versus patient privacy. Philos. Trans. R. Soc. A Math. Phys. Eng. Sci..

[238] Yang Q., Liu Y., Chen T., Tong Y. (2019). Federated machine learning: concept and applications. ACM Trans. Intell. Syst. Technol..

